# Speed Estimation for Visual Tracking Emerges Dynamically from Nonlinear Frequency Interactions

**DOI:** 10.1523/ENEURO.0511-21.2022

**Published:** 2022-05-12

**Authors:** Andrew Isaac Meso, Nikos Gekas, Pascal Mamassian, Guillaume S. Masson

**Affiliations:** 1 Department of Neuroimaging, Institute of Psychiatry, Psychology and Neuroscience, King’s College, London SE5 8AF, United Kingdom; 2 Department of Psychology, Edinburgh Napier University, Edinburgh, EH11 4BN, United Kingdom; 3 Laboratoire des Systèmes Perceptifs, Département d’Études Cognitives, École Normale Supérieure, Paris Sciences et Lettres University, Centre National de la Recherche Scientifique, Paris 75005, France; 4 Institut de Neurosciences de la Timone, Centre National de la Recherche Scientifique and Aix-Marseille Université, Marseille 13005, France

**Keywords:** dynamic nonlinearities, motion clouds, naturalistic stimulation, ocular following, probabilistic modelling, speed estimation

## Abstract

Sensing the movement of fast objects within our visual environments is essential for controlling actions. It requires online estimation of motion direction and speed. We probed human speed representation using ocular tracking of stimuli of different statistics. First, we compared ocular responses to single drifting gratings (DGs) with a given set of spatiotemporal frequencies to broadband motion clouds (MCs) of matched mean frequencies. Motion energy distributions of gratings and clouds are point-like, and ellipses oriented along the constant speed axis, respectively. Sampling frequency space, MCs elicited stronger, less variable, and speed-tuned responses. DGs yielded weaker and more frequency-tuned responses. Second, we measured responses to patterns made of two or three components covering a range of orientations within Fourier space. Early tracking initiation of the patterns was best predicted by a linear combination of components before nonlinear interactions emerged to shape later dynamics. Inputs are supralinearly integrated along an iso-velocity line and sublinearly integrated away from it. A dynamical probabilistic model characterizes these interactions as an excitatory pooling along the iso-velocity line and inhibition along the orthogonal “scale” axis. Such crossed patterns of interaction would appropriately integrate or segment moving objects. This study supports the novel idea that speed estimation is better framed as a dynamic channel interaction organized along speed and scale axes.

## Visual Abstract

**Figure:**
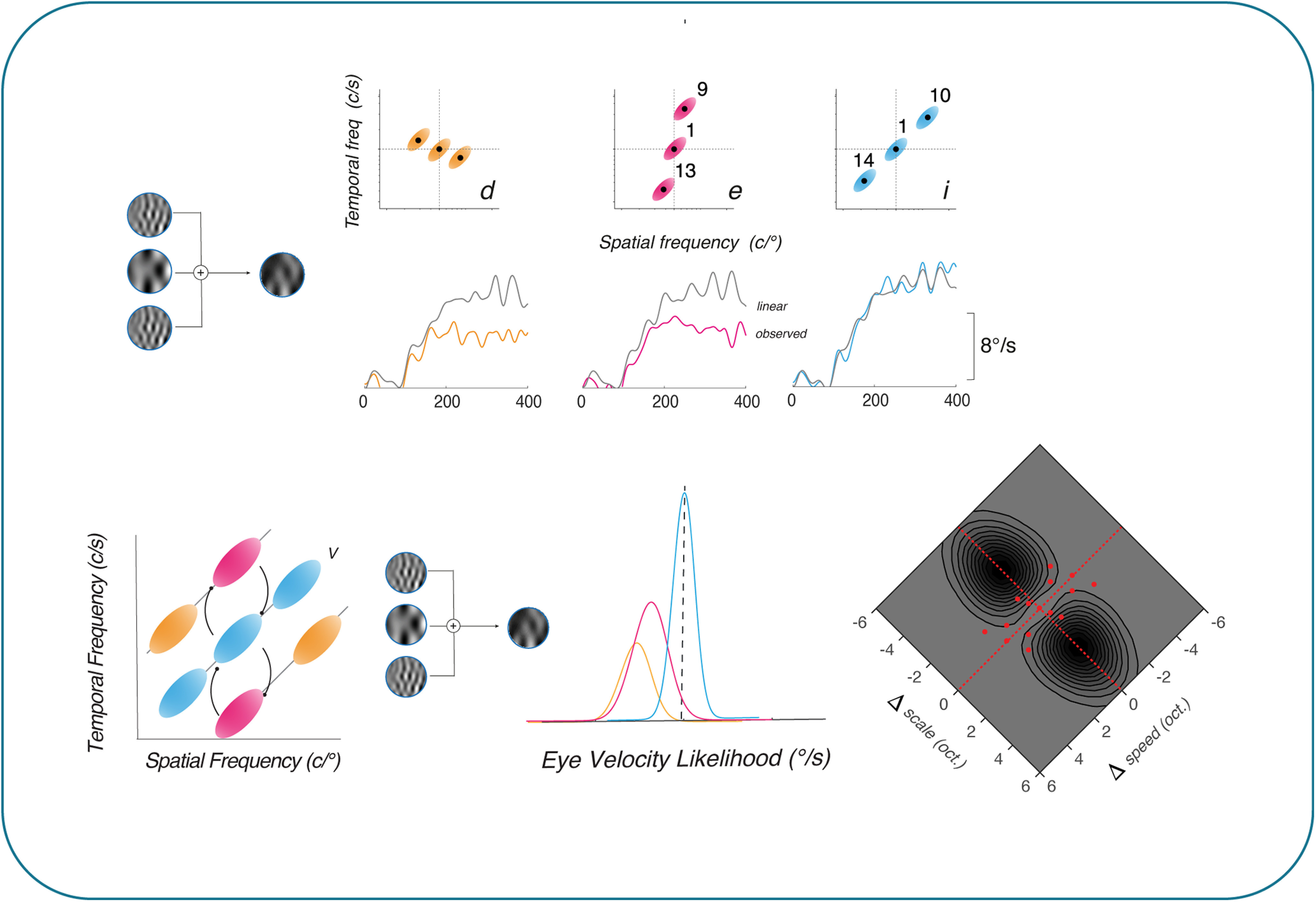

## Significance Statement

We presented participants with component moving luminance gratings or naturalistic moving textures with matched mean, but not variance, spatial and temporal characteristics. Recorded eye movements depended on stimulus type with more reliable speed estimates for naturalistic inputs. Eye responses to patterns made of several components evolved over ∼200 ms from linear to nonlinear information integration. The nonlinearity unveiled by our modeling work suggested concurrent inhibitory and excitatory processes acting within a motion representation that encodes stimulus speed (the ratio of temporal and spatial frequencies) and scale (the product of temporal and spatial frequencies) as key dimensions. This speed-scale framework recasts the classical spatiotemporal channel approaches to shed new light on motion integration and segmentation computations at both neuronal and behavioral levels.

## Introduction

Controlling actions in a dynamic environment requires the accurate measurement of trajectories of objects, obstacles, or animals estimated from retinal image motion. Therefore, visual systems within the animal kingdom probably share fundamental computational principles for measuring object direction and speed. In a cluttered environment, such motion computation is intertwined with another fundamental challenge of sensory processing: integrating or segmenting local information to parse the visual scene into behaviorally-relevant entities (Nishida et al., 2018).

The classical view is that motion information is computed within a two-stage hierarchical system corresponding to cortical areas V1-MT (Albright, 1984; Movshon et al., 1985; Movshon and Newsome, 1996; Born and Bradley, 2005). Local luminance energy is first sampled through primary visual cortex (V1) receptive fields acting as spatial and temporal frequency filters. Combining several of these filters generates motion opponency of direction-tuned cells. At the second stage, direction signals are nonlinearly combined to produce pattern direction cells that are insensitive to the orientation, contrast and spatiotemporal frequency properties of the image. In cortical Middle Temporal (MT) area, and subsequently, directions are uniformly represented (Kawano et al., 1994) and can be quickly decoded to drive smooth pursuit eye movements (Osborne et al., 2004).

While the same hierarchical network is used for human and nonhuman primate speed estimation, many aspects of speed processing remain unclear. In primates, speed tuned neurons were first thought to be present only at the integrative, MT stage (Perrone and Thiele, 2001; Priebe et al., 2003), but more recent studies have reported speed tuning in V1 complex cells (Priebe et al., 2006). Moreover, speed tuning is strongly affected by image properties such as contrast (Priebe et al., 2006) or spatiotemporal frequency content (Priebe et al., 2003; Krekelberg et al., 2006). Finally, retinal motion speeds are not uniformly represented in different cortical areas, with some speeds overrepresented during earlier encoding stages (Nover et al., 2005). These properties have perceptual and behavioral consequences. For instance, human and monkey tracking eye velocity and precision can be affected by first-order (e.g., spatial frequency, Priebe et al., 2006) and second-order (Simoncini et al., 2012; Mukherjee et al., 2015) statistics of inputs. Similarly, perceived speed can be biased by contrast, luminance, and frequency (Thompson, 1982; Smith and Edgar, 1990, 1994; Thompson et al., 2006). We recently reappraised how speed is estimated with moving patterns made of components tiling the spatiotemporal frequency space (Gekas et al., 2017). Using speed discrimination judgements, we unveiled a complex pattern of excitatory-inhibitory interactions between spatiotemporal frequency channels activated by the moving stimulus. It remains unknown whether these perceptual interaction patterns can be generalized to other speed-based behaviors.

Miles and colleagues coined the term “ocular following” for automatic, short-latency tracking responses to visual motion in human and nonhuman primates which reflect properties of low-level motion computation (Miles et al., 1986; Gellman et al.,1990). Tracking initiation is driven by a local motion energy computation followed by contrast normalization (Masson et al., 2001; Sheliga et al., 2005; Barthélemy et al., 2006). Ocular following is tuned for spatial and temporal frequencies (Gellman et al., 1990; Hayashi et al., 2010) and depends on local and global spatial center-surround integration (Barthélemy et al., 2006; Quaia et al., 2012; Sheliga et al., 2013). Successive linear and nonlinear 2D motion integration computations performed by MT and MST neuronal populations are reflected in its temporal dynamics (Masson and Perrinet, 2012).

**Figure 1.**
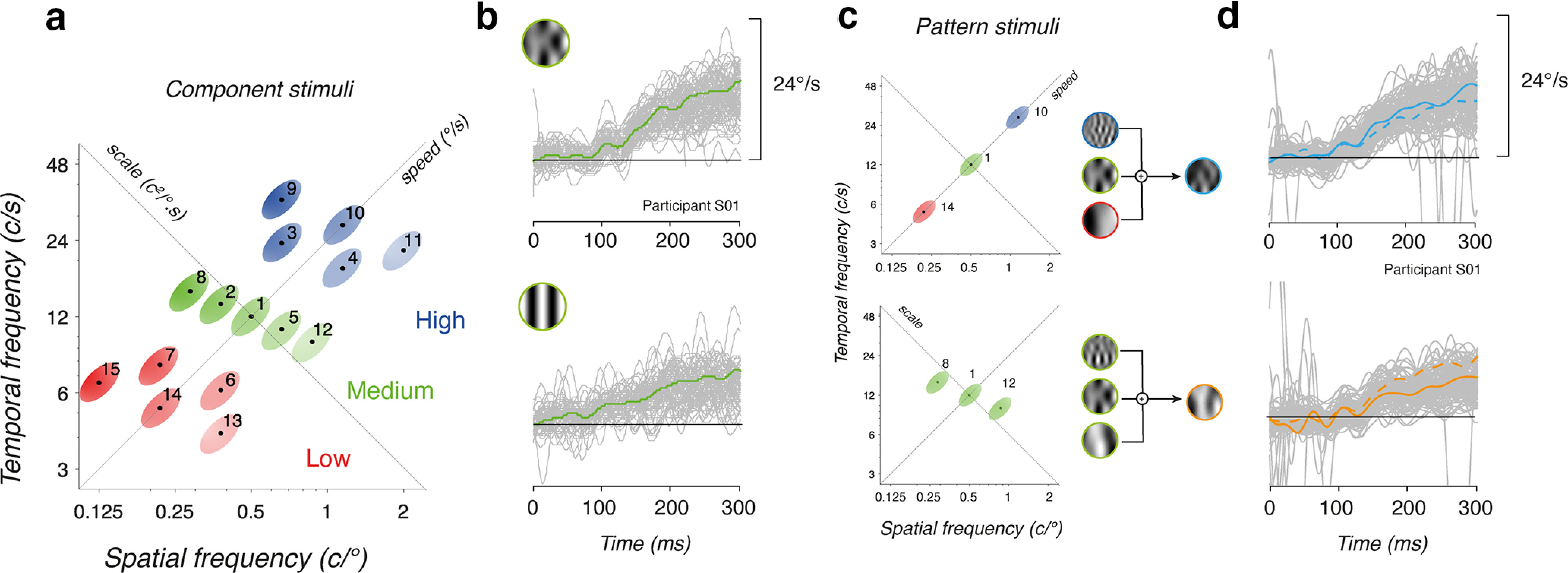
Rationale for probing motion integration properties for ocular tracking. ***a***, Motion stimuli are defined by their mean spatial and temporal frequencies in Fourier space. A set of 15 component stimuli were generated, paving the spatiotemporal frequency space (1 to 15 correspond to c1 to c15 detailed in Table 1). The component motion inputs were selected to cover three ranges of spatiotemporal frequency scales (low, medium, high, with a red-green-blue color code). They were also aligned (in groups of 3) along five different speed axes (from 11°/s to 53°/s, hue saturation code). ***b***, Ocular tracking responses to either a component Motion Cloud (MC, top) or a component Drifting Grating (DG, bottom) of same mean spatiotemporal frequencies and speed (stimulus c1 in panel ***a***). Gray curves are all single trials. Green curves are average across ∼150 valid trials, for participant S01. ***c***, Pattern stimuli were generated by summing several (2 or 3) of these components. For instance, a triplet can be oriented along the iso-velocity line or along the scale axis. Components were built from either component DG or component MC. Notice that both pattern stimuli have the same mean spatial and temporal frequency as well as the mean speed (here 24°/s). ***d***, Individual and mean eye velocity profiles of tracking responses to either pattern stimulus. Continuous blue/orange lines are the observed mean eye velocity profiles. Broken lines correspond to the linear prediction, computed as the mean of the responses to each component.

Our objective was to unveil dynamic patterns of interaction between spatiotemporal frequency channels that shape speed tuning. To do so we compared two types of motion component stimuli: simple drifting gratings (DGs) and motion clouds (MCs). Both had matched nominal spatial and temporal frequency (Fig. 1*a*), and MCs are additionally defined by their spatial and temporal frequency Gaussian distributions oriented along a given iso-velocity line within frequency space so that they efficiently drive tracking responses (Sanz-Leon et al., 2012; Simoncini et al., 2012). We unpacked the comparison in three main stages. First, we investigated the sensitivity envelope for each stimulus type. Next, we constructed moving patterns by summing two or three single DG or MC (Fig. 1*c*). We compared the tracking amplitude for patterns with a simple linear model averaging the corresponding component-driven responses. Last, deviations over time from linear integration unveil the dynamical pattern of interactions between spatiotemporal frequency channels (Gekas et al., 2017).

By completing the stages highlighted above, we make the following findings. (1) We demonstrate the importance of using well-parametrized naturalistic inputs, like MCs, in contrast to gratings, plaids, or random dot patterns. This naturalistic approach can be implemented from behavioral to physiological studies, in humans as well as vertebrate and invertebrate animal models. (2) We test the proposed novel theoretical framework, the speed-scale space, showing that it best captures observed interactions when two or three stimuli are combined into a pattern. This finding paves the way for a re-evaluation of visual motion segmentation and integration rules, as signaled in a recent review on visual motion computation (Nishida et al., 2018). (3) To complement the findings of Gekas et al. (2017), we show for the first time the generic applicability of this approach: perception and action (oculomotor) use the same nonlinear rules, albeit with the important elaboration of different excitation/inhibition kernels which in the present case emerge dynamically over the 400-ms stimulus presentation.

## Materials and Methods

### Participants

All experiments were conducted on six healthy volunteers (age M = 35.7, SD = 10.7, two females), four of whom were naive regarding the purpose of the study. Participants were free of neurologic and neuro-ophthalmological disorders, and all had normal or corrected-to-normal vision. The study was approved by the Aix-Marseille University Ethics Committee (Project SPEED, Ref 2014-12-3-06) and conducted in accordance with the principles of the declaration of Helsinki. Participants gave their informed consent before beginning the first session.

### Visual stimuli

Visual stimuli were computer generated offline with bespoke scripts in the MATLAB environment and stored as display matrices of 400-ms duration. Movies were displayed on an LCD display (CRS Display++, 1920 × 1080 pixels) at 100 Hz over a gray background of the same mean luminance (120 cd/m^2^). Stimulus presentation, eye movement recordings and online behavioral control were done on a Mac Pro computer running the PsychToolbox video libraries (Pelli, 1997) under the MATLAB environment. Two types of moving stimuli were used: broadband dynamic luminance noise MCs (Sanz-Leon et al., 2012; Gekas et al., 2017; Vacher et al., 2018) and drifting luminance gratings, DG which served as a narrowband (point in frequency space) control.

### MC stimulus generative model

MCs were used to systematically probe how luminance information which spans a range of spatiotemporal scales is integrated by the human visual system. MCs provide a generative model of naturalistic scenes constructed by a Primary Visual Cortex inspired basis set of dense localized drifting Gabor elements *G_i_* parameterized by characteristics *C_i_* (i.e., orientation *θ_i_*, spatial frequency *sf_i_*, and speed *v_i_* with corresponding temporal frequency *tf_i_*). These Gabor elements are linearly combined with randomized phases *φ_i_* to remove hard edges. An envelope distribution *E* is then applied to the characteristics of the elements *C_i_* in Fourier space to constrain the stimulus spread to an ellipse within the spatiotemporal frequency plane, see Figures 1. The resulting stimuli have a precise bandwidth controlled by parametric vectors *M* and *U* over orientation, spatial frequency, and speed:

(1) $$M=\left[\theta_{0},sf_{0},v_{0}\right]$$

(2) U=[Δθ,Bsf,Bv].

Where *M* in Equation 1 sets the center of the distribution of characteristics and *U* in Equation 2 their spread, the envelope distribution *E* is given by,

(3) E=P[M,U].

Which is a set of probability distribution functions parameterized by *M* and *U*. Orientation is defined by a Von Mises distribution, while spatial frequency and speed are Log Normal distributions. The characteristics *C_i_* of the Gabor’s *G_i_* are obtained by densely sampling the distribution *E*.

(4) $$C_{i}=\left[\theta_{i},sf_{i},tf_{i},v_{i}\right].$$

Each temporal frequency *tf_i_* is computed as the product of the spatial frequency *sf_i_* and the speed *v_i_*. To generate the two dimensional time varying stimulus *I*(*x,y,t*), a large finite number *N* of vector elements *G_i_* are each centered on image locations uniformly distributed across *p*[*p_x_*, *p_y_*] and defined by characteristics *C_i_*. The phase of each *G_i_*, *ϕ_i_* is uniformly distributed over [0, 2π]. The elements are all summed and scaled by an amplitude coefficient *a_o_* to control contrast and this generates an MC defined by its luminance *I* at each spatial location (*x*,*y*) over time *t*.

(5) $$I(x,y,t)=a_{o}I\left(x,y,t\right)=a_{0}\overset{N}{\underset{i=1}{\sum }}G_{i}\left(p_{i},\phi_{i}+t,C_{i}\right).$$

Equation 5 is implemented by an autoregressive AR2 process detailed in previous work, which is used to pregenerate the stimuli (Vacher et al., 2018). MCs used had five fixed parameters: *Θ_0_* = 90° (vertically oriented), *Δ*Θ_0_ = 15°, *B_sf_*=1 octave (full width at half max; FWHM), *B_tf_* = 1 octave (also FWHM) and RMS contrast = 60%. The variable parameters were the central spatial frequency *sf_0_* and temporal frequency *tf_0_*. which together determined the speed *v_0_ = tf_0_/sf_0_*.

**Figure 2.**
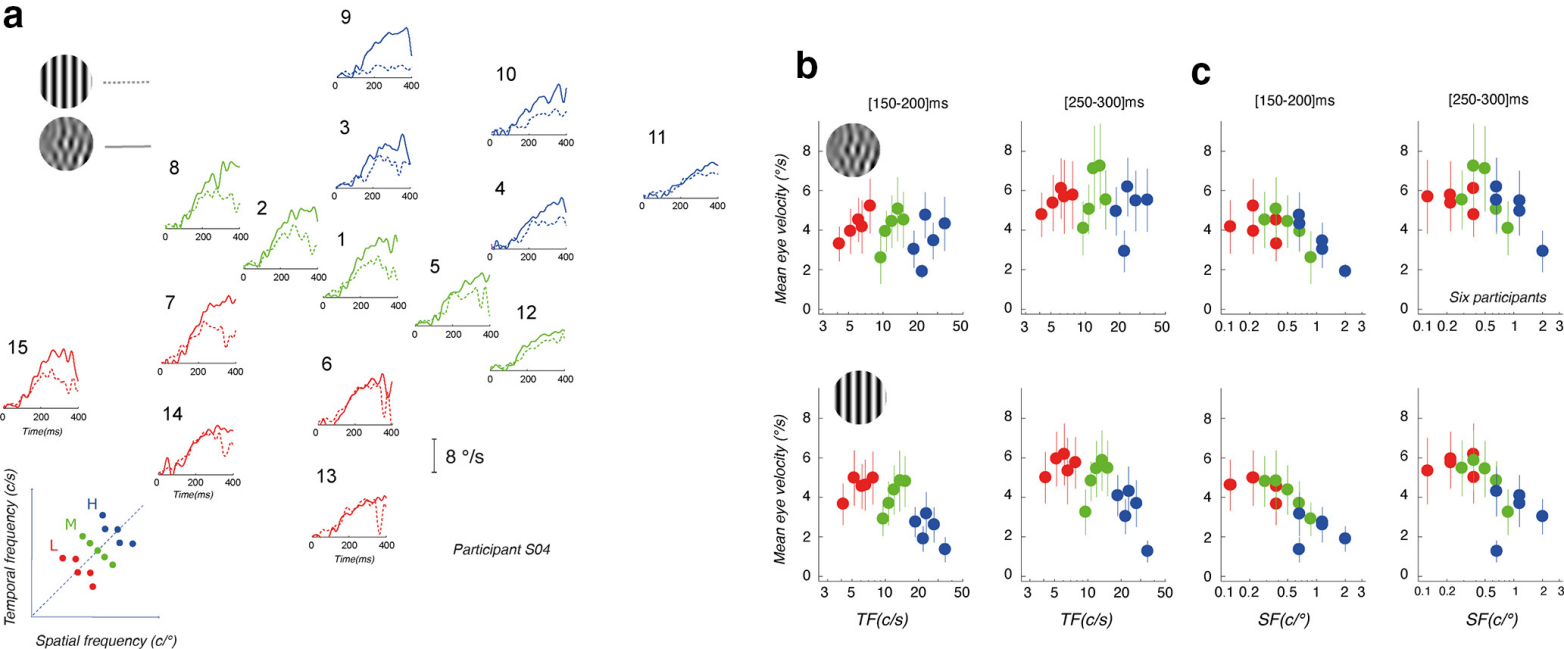
Dynamics of spatiotemporal frequency tuning of ocular tracking responses. ***a***, Mean eye velocity profiles of tracking responses to each of the component MC (continuous lines) or DG (dotted lines). At each location in the spatiotemporal frequency space, grating, and MC stimuli have identical mean spatial frequency, temporal frequency, and speed, but different spreads around these means. ***b***, Early (left) and late (right) temporal frequency (TF) tuning of ocular tracking responses to either component MCs (top) or DG (bottom). Data are mean (±SD) eye velocities across participants. ***c***, Same plots but for spatial frequency (SF) tuning.

**Figure 3.**
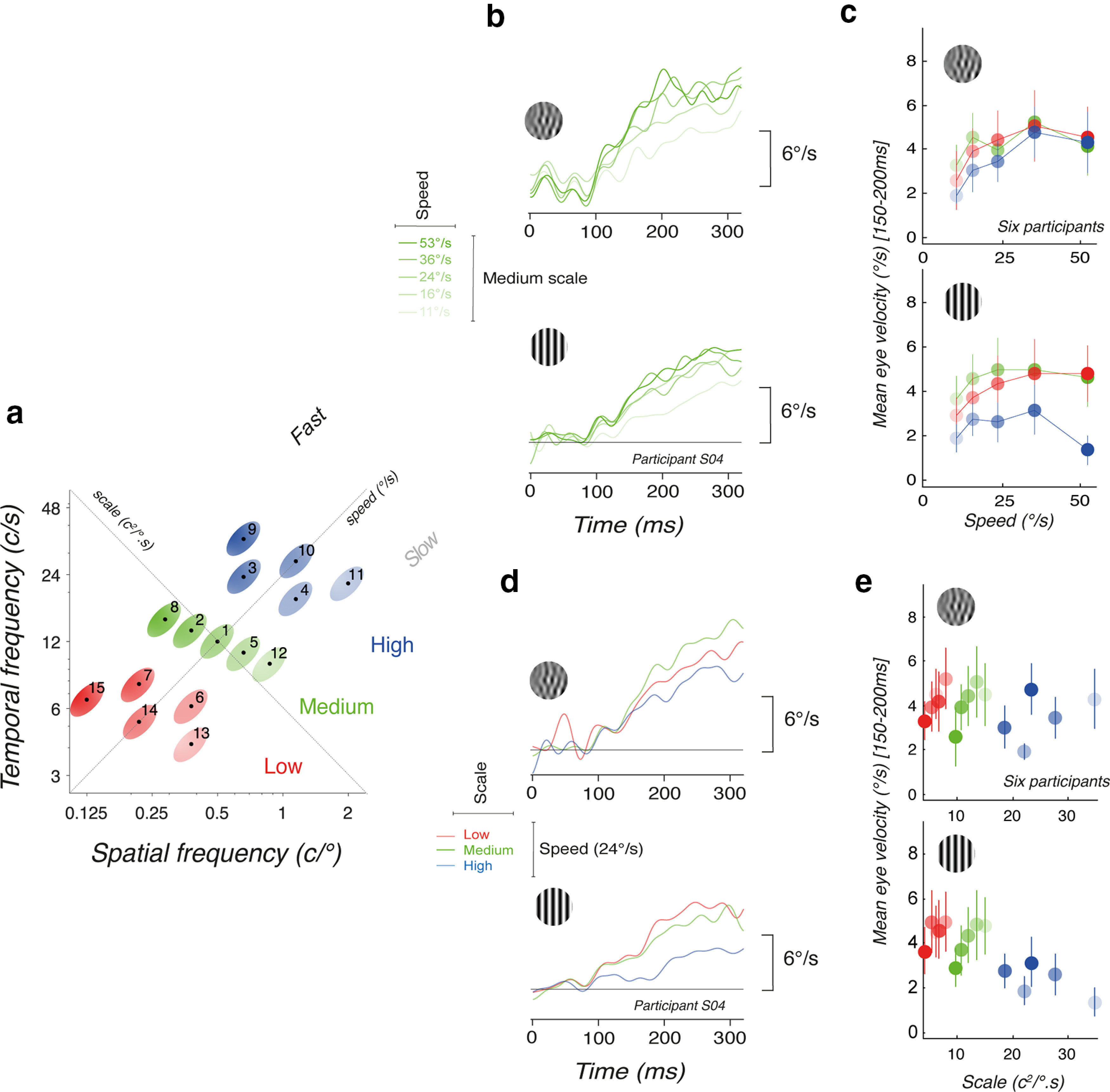
***a***, Distribution of 15 component DG and MC, relative to both the scale and speed axes (same as Fig. 1*a* with added labels for speed and scale). ***b***, Mean eye velocity profiles for component MC (top) and DG (bottom) of mean speed increasing from 11°/s to 56°/s, but sharing the same medium scale range. ***c***, Mean (±SD across participants) eye velocity, as a function of stimulus speed, for three different scale ranges. ***d***, Mean eye velocity profiles for component MC (top) and DG (bottom) of identical mean speed but increasing scale range. ***e***, Scale tuning of ocular tracking, when presented with either component DGs or MCs. Same color hue/saturation code.

Fifteen individual MC stimuli were generated with assigned numbers *c1* to *c15* arranged within the spatiotemporal frequency space as illustrated in Figures 1–3. These had a range of nine unique *sf_0_* values {0.13, 0.22, 0.29, 0.38, 0.50, 0.66, 0.87, 1.15, 2.00}c/° and 15 *tf_0_* values within the range [4.15, 34.69] Hz. These values were logarithmically spaced within the frequency plane to sample an obliquely oriented rectangular space of four octave range along the spatial frequency axis and three octaves along the temporal frequency axis, with each of the points arranged along one of five parallel oblique speed lines *v_0_* {10.96, 16.22, 24, 35.52, 52.58}°/s. Each MC stimulus was designed to target a distinct population of motion sensitive neurons, corresponding to a spatiotemporal/speed channel, which are limited in their frequency bands of sensitivity. The stimulus parameters for these 15 MCs are given in Table 1.

**Table 1:** Speed (v_0_), spatial (sf_0_), and temporal (tf_0_) frequency parameters of each of the MCs

MC #	Spd (°/s)	sf (c/°)	tf (Hz)	Relative distance (log)	Angle
c1	24.000	0.500	12.000	0.00	0.0
c2	35.522	0.379	13.459	1.00	157.5
c3	35.522	0.660	23.438	2.41	67.5
c4	16.215	1.149	18.626	3.13	27.9
c5	16.215	0.660	10.699	1.00	−22.5
c6	16.215	0.379	6.144	2.41	−112.5
c7	35.522	0.218	7.730	3.14	−152.1
c8	52.576	0.287	15.100	2.00	157.5
c9	52.576	0.660	34.690	3.66	75.4
c10	24.000	1.149	27.569	3.92	45.0
c11	10.956	2.000	21.911	5.04	23.5
c12	10.956	0.871	9.538	2.00	−22.5
c13	10.956	0.379	4.151	3.66	−104.6
c14	24.000	0.218	5.222	3.92	−135.0
c15	52.576	0.125	6.572	5.04	−156.5

The first MC, c1 lies at the left of the stimulus space and is used as the reference stimulus. Also included are the relative distances in log units and the polar angle from the reference c1.

### Control DGs generative model

Fifteen drifting luminance grating stimuli were used as comparison and control stimuli with the same spatiotemporal parameters as the MCs given listed in Table 1. These are parametrized using *M* in Equation 1 effectively implemented with zero valued distribution parameters *U* in Equation 2, generating a Dirac δ function at each point in the spatiotemporal plane. The orientation is the same as the MCs: *Θ_0_* = 90°. Each grating in which the phase is randomized by *Φ_0_* is given by *L* defined at each spatial location (*x*,*y*) and over time *t*.

(6) $$L\left(x,y,t\right)=A_{0}sin\left(sf_{0}.\frac{x}{2}.\pi +\phi_{0}+v_{0}.t\right).$$

The luminance contrast is scaled by the term *A_o_* and the spatial frequency *sf_o_* and speed *v_o_* for each individual component is given in Table 1.

### Composite stimuli: pattern MCs and pattern gratings

Dynamic eye responses to the 15 single stimuli (MCs and DGs) were measured. To understand the rules behind the visual system’s combination of individual components of different distances and orientations relative to the center of the spatiotemporal frequency space, we generated a set of nine pattern stimuli made up of three (or two) superimposed components from the set of 15 MCs and DGs. We linearly combined sets of such single stimuli into each of the patterns by adding components from Equations 5, 6 and then scaling them appropriately to get *F*.

(7) $$F(x,y,t)=b\overset{P}{\underset{j=1}{\sum }}I_{j}.$$

The summation of the *P* single stimuli uses *I* for each to generate pattern MCs (pMC) *F* as given in Equation 7 or alternatively uses *L* in place of *I* to generate *F* for the case of pattern DGs (pDG). Following summation, the result in each case is scaled by a coefficient *b* which like *a_o_* in Equation 5 adjusts the contrast to 60% ensuring all stimulus cases have a controlled perceived contrast. The nine patterns are identified by letters (a) to (i) and have parameters given in Table 2.

### Eye movement recordings and behavioral paradigm

Horizontal and vertical positions of the right eye were recorded with an EyeLink 1000 video-eye-tracker. Head movements were minimized by using a forehead and chin rest. Eye position signals were calibrated at the beginning of each block of 400 trials. Participants ran between three and eight blocks per day and data collection lasted several days. Experimental sessions were divided in two series, separating MCs and DGs stimuli. However, for each type of motion stimuli, all 15 components, nine patterns and a blank case were fully interleaved into blocks of 25 conditions. Experiments were ended after 10 blocks if about ∼150 valid trials were collected on each condition to ensure good signal-to-noise ratio and a sufficient sampling of eye velocity distributions for each condition. Overall, ∼8000 trials were collected on each participant (4000 each for MC and DG; 10 blocks of 400 trials).

We used the classical ocular following paradigm where motion stimulus directions were both unpredictable and presented in the wake of a centering saccade. This paradigm has been extensively used in both nonhuman (Miles et al., 1986; Masson et al., 1997) and human (Gellman et al., 1990; Masson and Castet, 2002; Simoncini et al., 2012) primates. A gray background was continuously presented to avoid large luminance fluctuations over the course of a block. At the beginning of a trial, a small, dark fixation target was presented at 11.6° eccentricity in the right hemifield, along the horizontal axis. Participants were required to fixate it within a 1° × 1° accuracy window. Once fixation was both accurate and steady for 100–200 ms, the fixation spot was turned off and a central fixation target was flashed. Participants had to make the 11.6°, leftward centering saccade to acquire it. The central target was turned off during the saccadic flight. Accuracy of the centering saccade was checked with an electronic window (2° × 2°). The moving stimulus was presented within a square patch (side: 18.6°) for 400 ms, starting 120 ms after the end of the centering saccade. At the end of the stimulus presentation, a gray background was displayed. The reappearance of the eccentric target indicated the start of a new trial. We interrupted on-line the trials where the centering saccade landed outside of the fixation window or when a second, corrective saccade was elicited. A blank trial was interleaved, where no visual motion was presented at the end of the saccade. It was used to subtract any postsaccadic drift that could contaminate eye velocity profiles. Motion stimulus trial conditions had the same probability as the blank. To avoid anticipatory tracking eye movements, both rightward and leftward motion directions were used, with the equal probability.

As explained, this centering saccade procedure has several advantages when investigating visual motion processing and reflexive tracking. First, the conditioning saccade guarantees that gaze will also be directed to the same location and therefore the retinal image will always be centered at stimulus motion onset. Second, visual motion sensitivity is boosted immediately after a saccade (Kawano and Miles, 1986; Gellman et al., 1990), a phenomenon called postsaccadic enhancement. Third, attention is most probably engaged at that central gaze location location and the fixation system disengaged given both the disappearance of the central fixation target and the short delay between saccade end and motion onset, similar to the classical gap paradigm (Krauzlis and Miles, 1996). Ocular following responses (OFRs) are reflexive, machine-like tracking responses that are elicited at short-latency (∼90 ms) by visual motion under these conditions. They are observed without specific instruction to the participants, beyond the instructed centering saccade.

### Data preprocessing and data analysis

Each block started after linearizing the right eye position with a nine-point calibration procedure. Rightward and leftward eye positions are given positive and negative values, respectively. Angular eye position signals were then filtered using a fifth-order Butterworth filter with a cut off at 40 Hz (3 dB) with a spline-interpolation to counter trace discontinuities. Velocity components were calculated by differentiating the filtered positions using a central difference method with position samples 10 ms apart to estimate a response every millisecond. Velocity was further filtered with a similar Butterworth filter with a cut off at 30 Hz. Rightward and leftward trials were aligned by inverting the sign of ocular responses to leftward visual motion trials. These eye data filtering and transforming procedures have been fully described elsewhere (Meso et al., 2016).

Eye velocity profiles of all trials were visually inspected using an interactive display and those with blinks, saccades and large oscillations within the 400 ms from stimulus onset of interest were removed. The frequency of these outliers varied across participants averaging ∼8% (4–16 exclusions per 160). The mean eye velocity of the blank trials was subtracted from the eye velocity profile of each selected trial to remove any residual postsaccadic drift (Gellman et al., 1990). For each condition, we computed the mean eye velocity profile to illustrate the dynamics of the ocular responses.

### Experimental design

The experiment had a multivariate and multifactorial repeated measures design with OFRs providing the main dynamic matrix of the dependent variables. OFR was analyzed either as high resolution pseudo-continuous traces with samples between 1 ms and 400 ms from onset at a 1 ms resolution or alternatively as binned responses over five successive 50-ms time windows between 50 ms and 300 ms. The first of these windows ran from [51–100 ms], covering the preresponse which can be used to estimate instrumental noise as a baseline. The next two were [101–150 ms] and [151–200 ms] covering the open-loop phase of tracking responses. The last two windows ([200–250 ms] and [250–300 ms]) covered the final part of the initial eye acceleration that we considered, as ocular responses tend toward the steady-state eye velocity (see Lisberger et al., 1981). In addition to this, other derived dependent variables such as orientation angles, Φ (speed) and Θ (scale), of the fitted spatiotemporal tuning surfaces and the linear/nonlinear ratio $R_{NL}=\left(e_{obs}/e_{pred}\right)$ of the pattern to the predicted component responses were also used for specific hypotheses. For each time window, and each valid trial, mean eye velocity was computed and then used as statistical measurements for response amplitude for a given time bin. All subsequent statistical analysis and modeling were performed using these eye velocity measurements as the dependent variables.

In our statistical testing, we had six stimulus or task factors of interest which were nine unique spatial frequencies (*sf_0_*), 15 temporal frequencies (*tf_0_*), two stimulus types (*stp*), the five time windows considered (*tw*), five speeds (*v_0_*) and 15 scales (*s_0_*) and an additional factor for the pattern stimuli, the spatiotemporal orientation angle (*oa*). The scales (*s_0_*) were calculated as the Euclidean distance from the origin [0,0] on the frequency axis along the reference iso-velocity line (v = 24°/s) to the point of intersection with the orthogonal line that cuts through the center of the stimulus of interest. We used six statistical models *M1* to *M6* to test our specific hypotheses. Five of these, *M1* to *M5* used linear mixed effects modeling. All these linear mixed effects models reported in this manuscript were conducted using an implementation of Satterthwaite’s method with adjusted estimates of degrees of freedom run with routines from the *lme4* and *lmertest* libraries in the programming language R (Bates et al., 2015; Kuznetsova et al., 2017). The Restricted Maximum Likelihood method (REML), was used for the fits to minimize the effect of the parameters which were not of interest. In each case, the independent variables of interest were included as the fixed factors while the random factors were selected to reflect factors we expected to vary the value of the DV as noise. The last statistical model *M6* was simply a nonparametric means comparison based on bootstrapping of 1000 samples of the linear-nonlinear separation times with trimmed means.

## Results

Figure 1 illustrates the rationale of the two series of experiments. Motion stimuli are classically defined in the spatiotemporal frequency space by their statistical properties such as mean spatial (*sf_0_*) and temporal (*tf_0_*) frequencies. We compared sinusoidal DGs to MCs. The former has a single spatial and temporal frequency and is represented by a point in this space. In contrast, MCs are broadband stimuli in Fourier space and therefore are defined by both their mean and SD of spatial and temporal frequencies. We defined our MC stimuli as being oriented, that is their energy is distributed along an oblique line and therefore are characterized by an oblique ellipse in Fourier space. Within the spatiotemporal frequency space, such an oblique axis defines an iso-velocity line, also named the speed axis. Along this line, all motion inputs have the same mean speed (and direction), but different spatial and temporal frequencies thus maintaining the same ratio (v_0_=sf_0_/tf_0_, in °/s). Gekas et al. (2017) proposed another axis to describe the spatiotemporal properties of motion input, called the scale axis. This axis is orthogonal to the speed axis (s=tf_0_*sf_0_, in c^2^/°.s). *Along this diagonal, inputs have different speeds but the same product of spatial and temporal frequencies*.

Our first goal was to investigate the properties of ocular tracking responses to a set of component DG or MC that covered a broad range of spatial and temporal frequencies (Fig. 1*a*). Mean spatiotemporal frequencies of the 15 motion components (labeled from *c1* to *c15*) were selected to tile the frequency space but also to probe different mean speeds (diagonal axis “speed”) and scales (diagonal axis “scale”). We separated these 15 components into three scale ranges (low, medium, and high, respectively, in red, green, and blue colors) and they were aligned along 5 different mean speeds, ranging from 11°/s to 52.6°/s. Figure 1*b* illustrates tracking eye velocities for ∼160 trials (all trials included in gray, but mean based on valid trials), in response to the central motion stimulus (*c1*, mean SF: 0.5 c/° mean TF: 12 Hz; mean speed: 24°/s), for either a MC (top) or a DG (bottom). Green continuous lines are mean eye velocity profiles. One can notice the difference in response amplitudes, initial eye velocity being larger for a component MC than for its DG counterpart, despite the fact that both stimuli have identical mean (but not distribution) spatiotemporal frequencies and speed. We will first describe the tuning properties of ocular tracking responses as a function of the statistical properties of the two motion inputs.

Our second objective was to probe the dynamics of ocular tracking in response to pattern stimuli constructed by summing different motion components. Figure 1*c* illustrates two examples for generating one pattern MC from three component MCs. In the top example, the three components (*c1*, *c14*, *c10*) are aligned along a single iso-velocity line in the spatiotemporal space (the speed axis), but span the three ranges of spatiotemporal scales. In the bottom example, another pattern MC is constructed by summing three components (*c8*, *c1*, *c12*) which are aligned along the same scale axis, but therefore span three different mean speeds. Notice that for both patterns, mean spatiotemporal frequencies and speeds are still identical. We recorded ocular tracking responses to these patterns, as illustrated in Figure 1*d*, where eye velocity profiles of single trials (gray) and average (continuous colored lines) are plotted. Broken lines illustrated the predicted mean eye velocity profiles, computed by averaging the responses to the three components presented independently. It is then possible to compare observed and linearly predicted responses to infer how visual inputs are combined, depending on their statistical properties, similar to what has previously been done for speed perception (Gekas et al., 2017).

### Spatiotemporal frequency tuning of ocular tracking

We first characterized the spatiotemporal tuning of ocular tracking responses with both stimulus types. Figure 2*a* illustrates mean eye velocity profiles, for a representative participant, of tracking responses for each component motion, for either a component DG (dotted lines) or component MC (continuous line). Overall, tracking responses are stronger with MC than DG. However, the difference between the two stimulus types arises later for higher temporal frequencies and speeds and is largely negligible for low temporal and spatial frequencies. We estimated the latency of component DG and MC-driven responses to 83.3 ± 9.4 ms and 88.1 ± 13.3 ms, respectively. There was no significant difference between the two types of stimuli. To quantify the tuning of ocular tracking to temporal and spatial frequencies, we computed mean eye velocity over five successive time windows of 50 ms each, binning the response dynamics from 50 to 300 ms after stimulus onset. The [50–100 ms] time window is a preresponse or baseline time window that allows us to estimate noise level just before response onset. Notice that the second and third time windows ([100–150 ms] and [150–200 ms]) of response corresponds to the initial open-loop phase of tracking (that is between ∼90 and ∼180 ms after stimulus onset) and together the response bins (from 100 to 300 ms) capture the overall eye acceleration phase as final eye velocity has not yet reached target velocity (Fig. 2*a*).

We first plot mean eye velocity (±SD across six participants) for early ([150–200 ms]) and late ([250–300 ms]) time windows, against temporal (Fig. 2*b*) and spatial frequency (Fig. 2*c*). For gratings, responses appeared to show a peak tuning across both stimulus dimensions, creating an inverted U-shape tuning function, peaking at ∼10 Hz with a broad temporal frequency tuning that depended on time window and stimulus type and low spatial frequency (peak at ∼0.16 c/° for DG and ∼0.30 c/° for MC). These tuning functions are present and measurable from tracking onset (125-ms window) for DGs and a little later for MCs and remain unchanged for later phases of tracking. Separating components based on their scale range does not change the overall spatial and temporal frequency tuning. With component MC, these tuning functions are broader and therefore less sharply visible, with limited changes in early response amplitudes when increasing either temporal or spatial frequency. Indeed, spatial frequency peaks could not be estimated reliably until the [150–200 ms] time window. For the late part of the responses, tuning functions are even flatter. Overall, tracking responses to gratings are more strongly tuned for spatiotemporal frequencies when those to MC are more subtly tuned to spatial and arguably untuned to temporal frequencies, at least not in a consistent way across our different scale ranges.

We quantified statistical dependencies in OFRs using mixed effects modeling. The first model, *M1* had OFR as the dependent variable predicted by fixed factors spatial frequency (*sf_0_*), temporal frequency (*tf_0_*), stimulus type (*stp*), time window (*tw*), and the interaction between *stp* and *tw* to compare temporal dynamics between component MC and DG. As a random factor, we used individual participants whose baseline eye velocity was expected to vary with stimulus type, over time windows and based on individual frequency functions. All linear mixed effects models reported in this manuscript are conducted using an implementation of Satterthwaite’s method run with routines from the *lme4* and *lmertest* libraries in the programming language *R* (Bates et al., 2015; Kuznetsova et al., 2017). Further details are given within Materials and Methods.

There were main effects of *sf_0_* (*F*_(6.3)_ = 9.47, *p* = 0.020) and *tw* (*F*_(7.3)_ = 5.14, *p* = 0.028). There was no significant effect of temporal frequency (*tf_0_*) or stimulus type with *p* > 0.05. There was however a strong interaction between the time window and stimulus type (*F*_(867.8)_ = 13.69, *p* < 0.001). Looking at the pattern of this interaction, the fitted statistics was *tw* > 4 for windows 3–5 (*p* < 0.01), indicating that MC responses became gradually stronger than DG responses over the duration of the response, with a significant difference between MCs and DGs from 175 ms onwards. We did not explore the significant spatial frequency tuning further with the linear mixed model, but instead did so with surface fitting.

### Speed and scale tuning of ocular tracking responses

Since mean spatial and temporal frequencies of the motion component cannot explain responses tuning for both DG and MC together, we investigated how amplitudes would be affected by either the input mean speed or scale properties. The set of 15 component stimuli are replotted in Figure 3*a*, together with labels for the speed and scale axes. Recall that spatiotemporal frequencies are grouped within three scale ranges (color hue) and were distributed along five different speeds (11–53°/s), indicated by color saturation.

Figures 3*b*,*c* illustrate speed tuning of ocular responses. For both component MC and grating, we plot mean eye velocity profiles for a subset of five conditions (*c8*, *c2*, *c1*, *c5*, *c11*), where all motion stimuli belong to the medium scale range but span the whole speed range. Amplitude of ocular responses clearly increases with higher speeds (Fig. 3*b*), in particular for the later parts of the responses (>150 ms). Amplitudes of speed modulations are larger with component MC than DG. Figure 3*c*, upper plot, plots the mean (±SD across six participants) eye velocity, as a function of target speed, for the three scale ranges, for the early time window ([100–150 ms]). With component MC, speed tuning was independent of stimulus scale: all three curves were superimposed, peaking at ∼30°/s. By comparison, the lower panel shows that speed tuning was mostly flat for component DG, and strongly different for stimuli with the highest scales (blue symbols). In particular, with the highest scale stimuli (see stimuli *c10* and *c11*), responses to gratings were strongly reduced, when compared with MC despite their identical mean spatial and temporal frequency contents.

Next, we ordered the tracking responses according to stimulus scale. Figure 3*d* plots, for the same participant, the mean eye velocity profiles obtained with three component MC (top) and grating (bottom) stimuli of the same speed (24°/s) but different scales (*c14*, *c1*, and *c10*). Again, ocular tracking responses were slightly modulated by scale range for MC, but strongly reduced for highest scales with DG (blue curves). Early mean eye velocity is plotted against scale in Figure 3*e* (mean ± SD across six participants) for both component MC (upper plot) and DG (lower plot). While tracking initiation is only marginally tuned for scale, across speed ranges, there is a clear low-pass scale tuning with component DG. We observed the same pattern of scale tuning at later time windows.

These results suggest that ocular responses are tuned for either speed or scale depending on stimulus type. To statistically test for these effects, we used a second mixed model *M2* with a similar structure to *M1* used above but substituting the frequency factors *st_0_* and *tf_0_*. For *M2*, binned eye velocity was predicted by fixed factors scale (*s_0_*), speed (*v_0_*), *stp*, and *tw* and again the interaction between stimulus type and time (*stp,tw*). The random factor was individual participants, with baseline tracking eye velocity being dependent on *stp*, *tw* and individual frequency response functions. There are main effects of speed (*F*_(5.4)_ = 6.38, *p* = 0.049), scale (*F*_(5.2)_ = 17.73, *p* = 0.0076) and time window (*F*_(7.3)_ = 4.91, *p* = 0.031). Stimulus type alone however does not show a significant effect, *p* > 0.05. Once again the interaction (*stp,tw*) is significant (*F*_(867.9)_ = 13.22, *p* < 0.001), suggesting that differences emerged over time and that the strong tuning effects observed for *s_0_* and *v_0_* are driven primarily by MC in the later time windows.

Overall, results shown in Figures 2, 3 reveal that ocular responses are strongly modulated by both spatiotemporal frequency and scale contents when presented with point like (nonoriented) motion stimuli (DG) but are possibly more reliably tuned for speed with oriented motion inputs (MC). Thus, speed tuning to MC may be less sensitive to both mean spatial and temporal frequencies properties of the inputs and more strongly dependent on stimulus speed. We explore this difference in tuning further in the next section.

### Temporal dynamics of spatiotemporal, speed, and scale tuning functions

We have shown above that tuning functions of ocular tracking for the different stimulus parameters can evolve over time such that while the overall response amplitude broadly increases, different inputs can drive specific changes in the shapes of early or late phases of tracking. Spatiotemporal tuning surfaces have been previously computed for human and monkey ocular following using 2D Gaussian models (Hayashi et al., 2010; Miura et al., 2014). However, we show above that spatial and temporal frequency tunings are not always independent and that, with our limited set of conditions, temporal tuning is poorly described by a Gaussian function. Therefore, we fitted a more assumption free, 2D polynomial function (quadric surface) through the 15 response amplitudes for each participant as well as for the mean values across participants, from the earliest ([100–150 ms]) to the latest ([250–300 ms]) phases of tracking responses. Such a simple model nicely renders the spatiotemporal tuning of tracking behavior, with adjusted *r*^2^ correlations ranging from 0.9 to 0.96 across both component DG and MC data.

**Figure 4.**
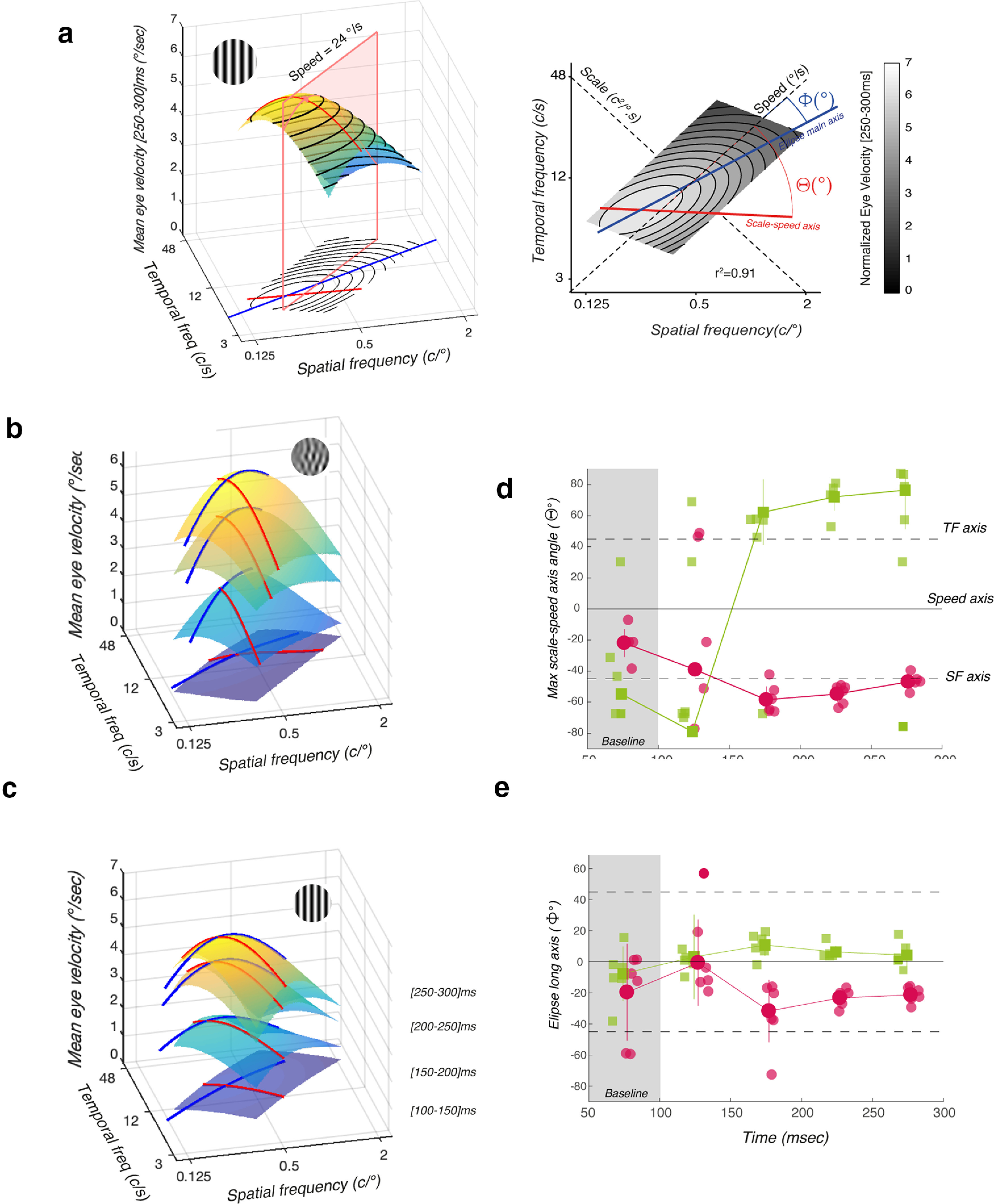
Temporal dynamics of ocular tracking tuning. ***a***, Spatiotemporal tuning of response amplitude computed across the 15 component DG for a given temporal window (here [250–300 ms]). The spatiotemporal tuning was approximated by a quadric surface (surface plot on the left, contour plot on the right). From these quadratic fits, we extracted the main axis (blue curve) that tracks pairs of spatiotemporal frequency coordinates along which the amplitude changes the least for each scale within the fitted range. We also estimated the maximum scale-speed axis (red curve) by collecting the scale corresponding to the maximum eye velocity for each stimulus speed. ***b***, Best fitted spatiotemporal tuning surfaces for component MC stimuli computed across six participants and for four successive time windows, from [100–150 ms] to [250–300 ms] after stimulus onset. ***c***, Same as in ***b*** but for component DG stimuli. ***d***, For each participant, the angle Θ of the maximum scale-speed axis is plotted for five time windows, for both DG (pink) and MC (green) stimuli. Larger dots with error bars show mean ± SD (across participants). The first time window is indicated as baseline, before response onsets. ***e***, For each participant, the angle Θ of the main axis of the spatiotemporal tuning surface is plotted for five time windows.

Figure 4*a* shows a 3D rendering of one best-fit spatiotemporal surface (left panel), together with its 2D projection (right panel), for mean eye velocity (across six participants), at the latest time window. From this surface, we extracted several properties. The red line corresponds to the scale values yielding the largest responses, at each stimulus speed. We can compute the angle *Θ* between this max-speed-scale axis and the speed axis. When *Θ* = 90°, this line coincides with the scale axis. The long axis of the envelope is plotted in blue, with its angle relative to the speed axis being denoted *Φ*. When *Φ* = 0, the main orientation of the spatiotemporal tuning function is aligned with speed. When *Φ* = 45 or −45, the tuning function is aligned with temporal or spatial frequency, respectively.

Figure 4*b* plots these best-fit spatiotemporal surfaces at different time windows, for either component DG or MC. With DG, the spatiotemporal envelope exhibits the classical low-pass properties already described by others for OFRs (Miles et al., 1986; Gellman et al., 1990; Hayashi et al., 2010). Over time, the orientation of the envelope remains stable, with its main axes being closer to spatial and temporal frequency axes than speed or scale. Moreover, optimal spatiotemporal frequencies are remarkably stable over time. By contrast, the envelope observed with single MCs gradually rotates over time such that, at the latest time window, it was mostly tuned for speed rather than spatial or temporal frequencies. Accordingly, the max-speed-scale line for MC was closer to the scale axis at the end of the tracking.

We then computed *Θ* and *Φ* angles for each participant and time window. Figure 4*d* plots the individual (and mean ± SD, continuous lines) angles *Θ*, from 75 to 275 ms after stimulus onset. For DG, in Figure 4*c*, the *Θ* angle is remarkably constant at ∼−45°, indicating that spatial frequency mostly determined the maximum amplitude at a given input speed. With component MC, this axis rotates from ∼−60° to ∼+80°, indicating that scale properties set maximum response amplitude at a given speed. These dynamics were similar across participants, as illustrated by the small SD values. Accordingly, the main axis of the spatiotemporal tuning (angle *Φ)* was steadily close to 0 for MC but rotated toward ∼−30° for DG. Thus, over time, spatiotemporal tuning for MC is best described by the speed and scale axis than by spatial and temporal properties. With DG, and with our conditions, spatiotemporal tuning is best described by spatial and temporal factors independently.

To quantify this dynamic shift in the angles *Θ* and *Φ* relative to speed and scale axes we used a third mixed effects model *M3*. We run a linear mixed effects model with tuning angle *Θ* (Fig.4*a*,*d*) predicted by the fixed factors *stp* and *tw* and their interaction (*stp,tw*). As a random factor were participants, each with a tuning angle which depended on individual baselines, stimulus type and window dynamics. The main effect of stimulus type was significant with component MCs having an angle *Θ* that was larger by an average 71.6° and better aligned with the speed axis than with component DG (*F*_(45.3)_ = 6.58, *p* = 0.0137). There was no main effect of the time window (*F*_(5.4)_ = 4.60, *p* = 0.081). There was however a significant interaction (*stp,tw*) (*F*_(50.2)_ = 26.2, *p* < 0.001). This interaction confirms what can be seen in Figure 4*d*, indicating that the MC angle is separated from the DGs from the 175-ms window onwards. We additionally run a similar test for the angle of the major axis of the fitted ellipse *Φ* which represents the scale axis. This angle *Φ* was also predicted by fixed factors *stp*, *tw*, and their interaction (*stp,tw*). As random effects, we had participants with individual baselines dependent on stimulus type and windows. There were no significant main effects for stimulus type (*F*_(45.6)_ = 0.46, *p* = 0.503) or time window (*F*_(5.5)_ = 0.019, *p* = 0.90).

Finally, for the sake of comparison with previous studies, we carefully compare our 2D polynomial function with the spatiotemporal tuning previously described using the 2D Gaussian function of Log(*sf_0_*) and Log(*tf_0_*). First, we note that around the peak of the envelope, the best fit polynomial parameters can be numerically related to best-fit spatial and temporal frequency means of a 2D Gaussian function. One can relate *Θ* and *Φ* angles to the (in)separability of spatial and temporal frequency tuning as previously reported for ocular following in humans (Hayashi et al., 2010; Sheliga et al., 2016) and monkeys (Miura et al., 2014). In particular, a coefficient *Q*, one of the parameters from the Log Gaussian fitting of spatiotemporal frequencies, has classically been used as an index of the speed tuning of neural or behavioral responses (Priebe et al., 2003, 2006; Miura et al., 2014). With this classical approach, the spatiotemporal frequency surface function contains two Log Gaussians corresponding to separate spatial and temporal frequency tuning terms. *Q* acts as a linear factor inside the exponent of the temporal frequency term, determining the weighting of a spatial frequency tuning contribution to that term. The equations can be configured with a structure such that if *Q* = 0, then the channels are speed tuned (i.e., spatial and temporal frequency tuning are inseparable), whereas if *Q* = −1, then the frequencies are independently coded or separable (Priebe et al., 2006). This would correspond to ellipses of spatiotemporal tuning that are either oriented (along the speed axis) or more circular. It can be shown from partial first and second derivatives of the surface functions that *Q ∼* −(1 + *b_4_/2b_5_*) where *b_4_* and *b_5_* are the coefficients of the f_x_*f_t_ and the *f_t_^2^* terms, respectively, in our polynomial fit (see Eq. 12).

**Figure 5.**
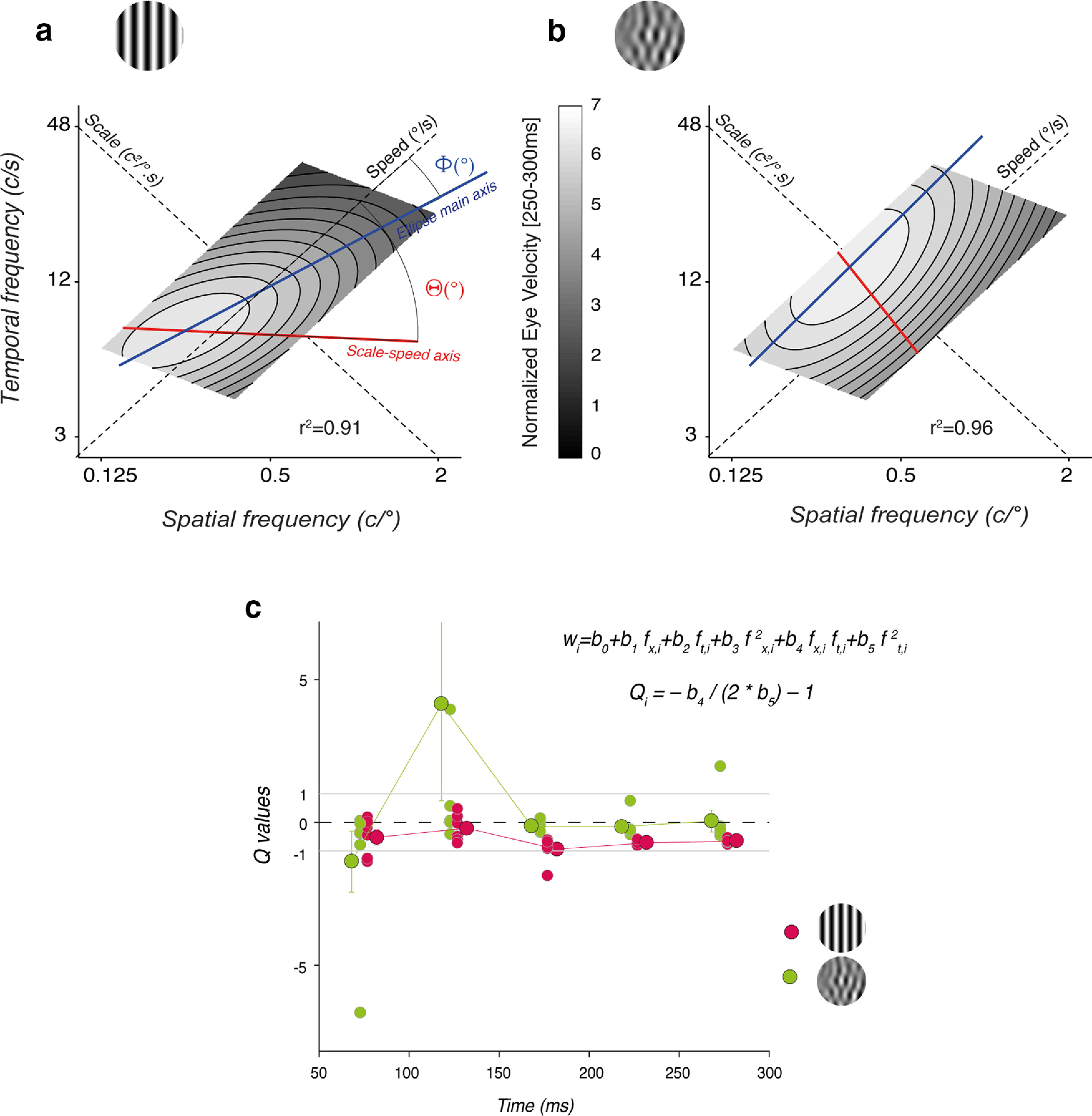
***a***, ***b***, Best fit 2D polynomial function describing the relationship between ocular following amplitude and both spatial and temporal frequencies. Data are mean response amplitudes across six participants. ***a***, Component DGs. ***b***, Component MCs. ***c***, Q index can be derived from the best-fit 2D polynomial functions, as indicated with the two equations. Individual Q indices were computed for each participant, and each time window. Individual and mean (±SD, across participants) are plotted. Note that one outlier (MC condition) with a Q value of ∼22 at time 150 ms is not plotted to allow comparison between the two conditions.

From this analysis, we show that optimal spatial and temporal frequency tuning given by either 2D Gaussian and polynomial fits are consistent. More important, Figure 5 shows that *Q*, as estimated from the best-fit individual polynomial functions is around 0 for MC and around −1 for DG, consistent with the *Φ* estimates and confirming that with MC spatial and temporal frequency tuning are inseparable while, when tested with DG, ocular following tuning is best described with independent spatial and temporal frequency functions (Hayashi et al., 2010; Sheliga et al., 2016).

### Ocular responses to component MC are more reliable

**Figure 6.**
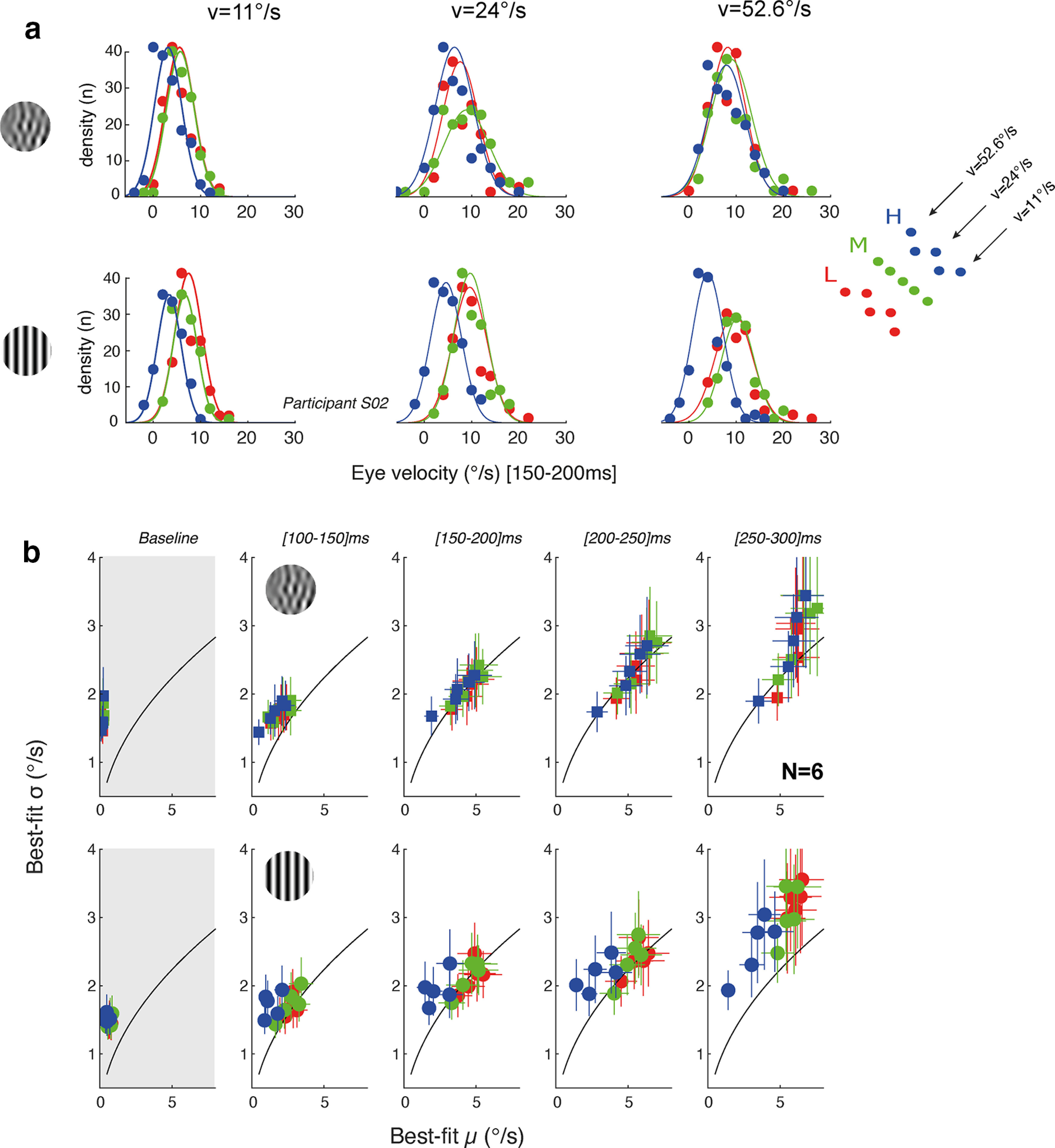
Variability across trials of tracking responses. ***a***, Distributions of early eye velocities, for participant S02. Data are shown for three mean speeds (from left to right) and for each speed, with three different spatiotemporal frequencies and therefore three different scale ranges (color code). Motion stimuli were component MCs (top) or DGs (bottom). Continuous curves are best-fit Gaussian functions. ***b***, Relationship between best-fit parameters σ and μ. Data are mean (±SD) across six participants for each of the 15 motion components, for either MCs (top) or DGs (bottom). Color code indicates the scale range of each data point. From left to right, plots illustrate results obtained for the five successive time windows. The first time window ([50–100 ms], gray shaded) provides an estimate of baseline variability, as most of this measurement window covered preresponse time. Continuous black lines indicate the theoretical relationship where σ increases with the square root of the μ value.

Several previous studies, at neuronal (Priebe et al., 2006) and behavioral (Osborne et al., 2007; Simoncini et al., 2012) levels have suggested that the statistics of visual motion inputs strongly impact response variability across trials. Figure 6*a* illustrates, for one participant, frequency distributions of mean eye velocity, across trials, for three different mean stimulus speeds (from 11°/s to 52.6°/s). Each set of data points corresponds to one of the three different spatiotemporal frequency scale ranges, estimated for the early ([150–200 ms]) phase of tracking responses. Distributions were fitted with a Gaussian function. As mean speed increases, distributions shift to the right, corresponding to faster eye velocity during tracking initiation, for both component MC and DG. However, for any given stimulus speed, the distribution overlaps and does not change in width with MC. On the contrary, with DG, distributions shift toward lower eye velocities, and broaden when scale increases. In particular, responses to the highest scale range (blue symbols) were both smaller and more variable.

Thus, different motion inputs of the same speed appear to drive ocular tracking responses with different precision. Moreover, over the whole observed time lapse, tracking continuously accelerates toward target motion speed. Increasing eye velocity would naturally increase its variability within and across trials. This noise-amplitude relationship would be expected to lead to a theoretical square root relationship between *μ* and *σ* parameters of the Gaussian functions fitted to the eye velocity distributions across trials, for each time window as signal thresholds have been shown to be limited by shot noise at the sensory input stages (Osborne et al., 2007). These relationships are illustrated in Figure 6*b*, for each time window. Notice that the leftmost plot corresponds to the baseline time window, just before response onset ([50–100 ms]), as used to estimate noise during fixation (Osborne et al., 2004). Symbols are mean ± SD values (across six participants) of these parameters, for each of the 15 components and each stimulus type. The continuous black lines indicate this theoretical square root scaling. Before 200 ms (relative to stimulus motion onset), variability and amplitude varied according to this simple model. After 200 ms, variability of ocular responses increased more than predicted for component DG, regardless of the scale range. This result confirms that ocular tracking responses to component DG were more variable than to component MC, in particular for the late phase of tracking initiation, that is later than 200 ms after stimulus motion onset.

To statistically contrast the dynamic relationship between variability and response for the two stimulus types, we use our fourth statistical model *M4*. The difference between the SD *σ* empirically measured for the tracking responses for each participant across trials and that predicted from the square root of the mean response μ, was calculated as a dependent variable $\sigma_{d}=\sigma -\sqrt{\mu }$. This estimated value should be approximately zero if stimuli show a $\sqrt{\mu }$ relationship between variability and average response. In model *M4*, the relationship between the DV, *σ_d_*, and fixed factors *tw*, *stp*, and scale *s*, and three interaction terms, (*spt,tw*), (*s,stp*), and (*s,tw*), were used. As random factors, we used the individual participants with a baseline value adjusted by stimulus type. There were main effects of time window (*F*_(880)_ = 18.29, *p* < 0.001), stimulus type (*F*_(12.4)_ = 13.63, *p* < 0.01) and scale (*F*_(880)_ = 3.20, *p* = 0.0412), indicating that all of these factors significantly changed the mean response and variability relationship. There was also a strong significant interaction between the time window and stimulus type (*F*_(880)_ = 40.79, *p* < 0.001). Further inspection showed that MC differences were closer to the $\sqrt{\mu }$ prediction at later time windows. There was also a significant interaction between stimulus type and scale (*F*_(880)_ = 4.38, *p* < 0.05), but no significant interaction between time window and scale (*p* > 0.05). Thus, responses to MC were less variable than those to component DG when taking into account the expected variability from response amplitude. This is true in particular for the highest scale.

**Figure 7.**
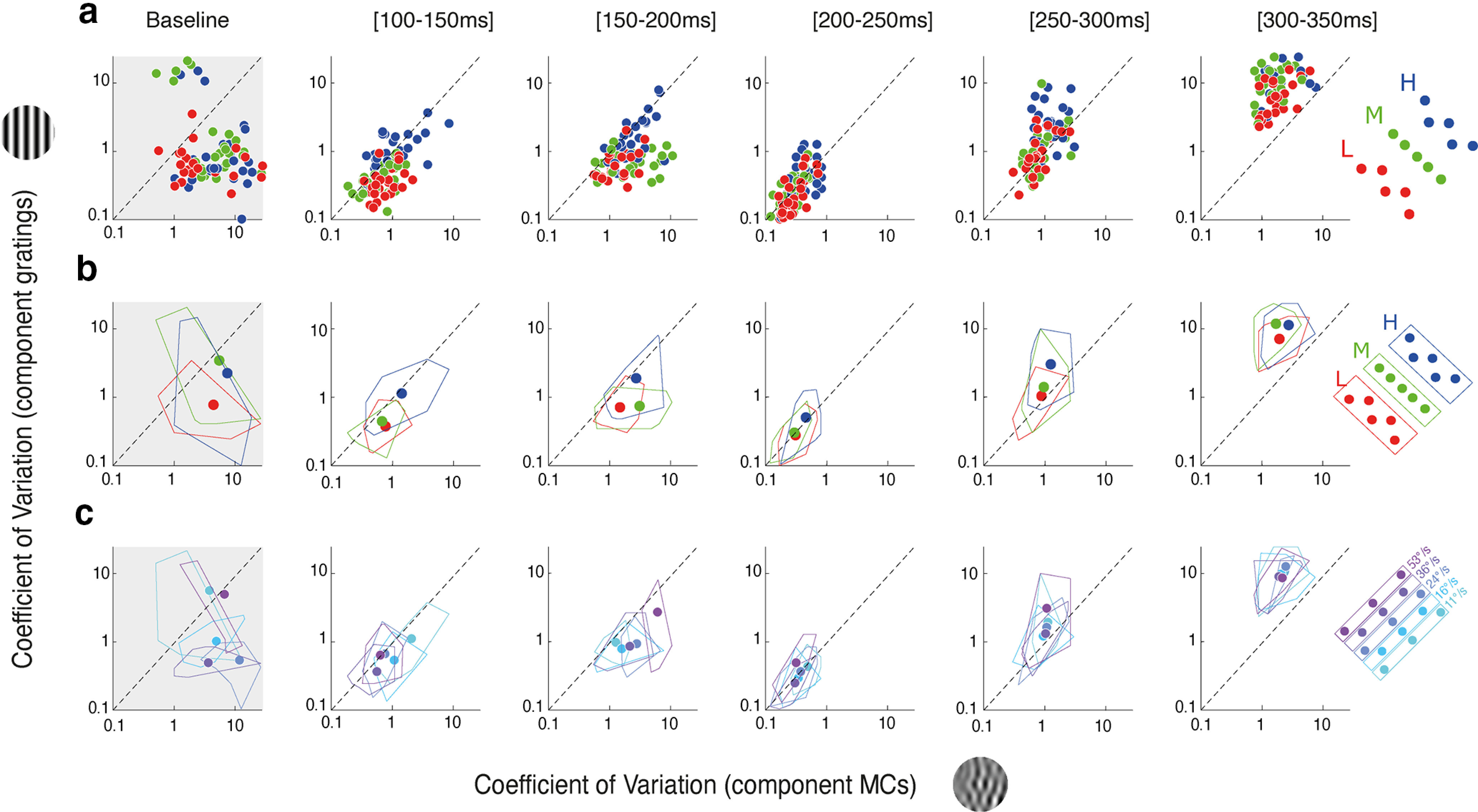
Speed or scale tuning of tracking responses variability. CV values estimated for ocular responses to component MCs or DGs are plotted one against the other, for six time windows, starting at 50 ms (left column) and spanning 50 ms each. The first time window ([50–100 ms]) estimates the relative variabilities over a baseline, preresponse epoch (gray shaded). ***a***, Each dot corresponds to a given component stimulus pair, for all participants. Colors correspond to the scale range. ***b***, CV values are averaged across speeds, and participants, for each of the three scale ranges. Continuous contour lines enclose the variance across participants. ***c***, CV values are averaged across scales, and participants, for each of the stimulus speeds.

Analyzing the relationships between mean (*μ*) and variance (*σ*) of eye velocity distributions suggest that higher scales (i.e., higher spatial and temporal frequencies; Fig. 6*a*,*b*, blue symbols) more strongly impaired behavioral reliability when tracking component DG, as compared with MC. Therefore, we checked whether such an increase in variability could be tuned for either scale or speed by pooling data across either speeds or scales. To do so, we computed the coefficient of variation (CV=σ/μ) to compare response variability, after normalizing by response amplitude. To compare across stimulus types, we plot CV values obtained with component DGs as a function of the corresponding component MCs (Fig. 7). In Figure 7*a–c*, data above the diagonal unity lines indicate that variability of responses to DGs is larger than for a MC, for the same condition and regardless of their relative amplitude. Looking at shifts in data point positions within the plots relative to the diagonal line of unity as we go from left to right panels is indicative of stimulus-dependent dynamic shifts in response variability. We tracked these relationships across the five time windows, binning the responses from 100 to 300 ms after stimulus onset. Again, a baseline time window ([50–100 ms]) is shown to estimate noise before response onset.

Figure 7*a* shows that variability of responses to component DG was larger than observed with MC, for the late time windows (>200 ms) but not the earlier ones. This observation holds for all scale ranges (Fig. 7*b*). In fact, relative variability was larger with MCs over the first time windows, corresponding to tracking initiation. To further illustrate how variability changes with scale and speed ranges, we average CV over the five components defining one scale range. Similarly, we computed CV values for the five mean speeds independently, pooling data across scale ranges (Fig. 7*c*). The same pattern of dynamics is visible: variability was larger for MC than DG at tracking initiation but then reversed and became larger for DG than MC for the late part of tracking responses, across all speeds. At the closing of the open-loop phase, corresponding to the [200–250 ms] time window, responses to either component DG or MC show the same variability, across both scale and speed ranges.

### Ocular tracking responses to pattern motion speeds

**Figure 8.**
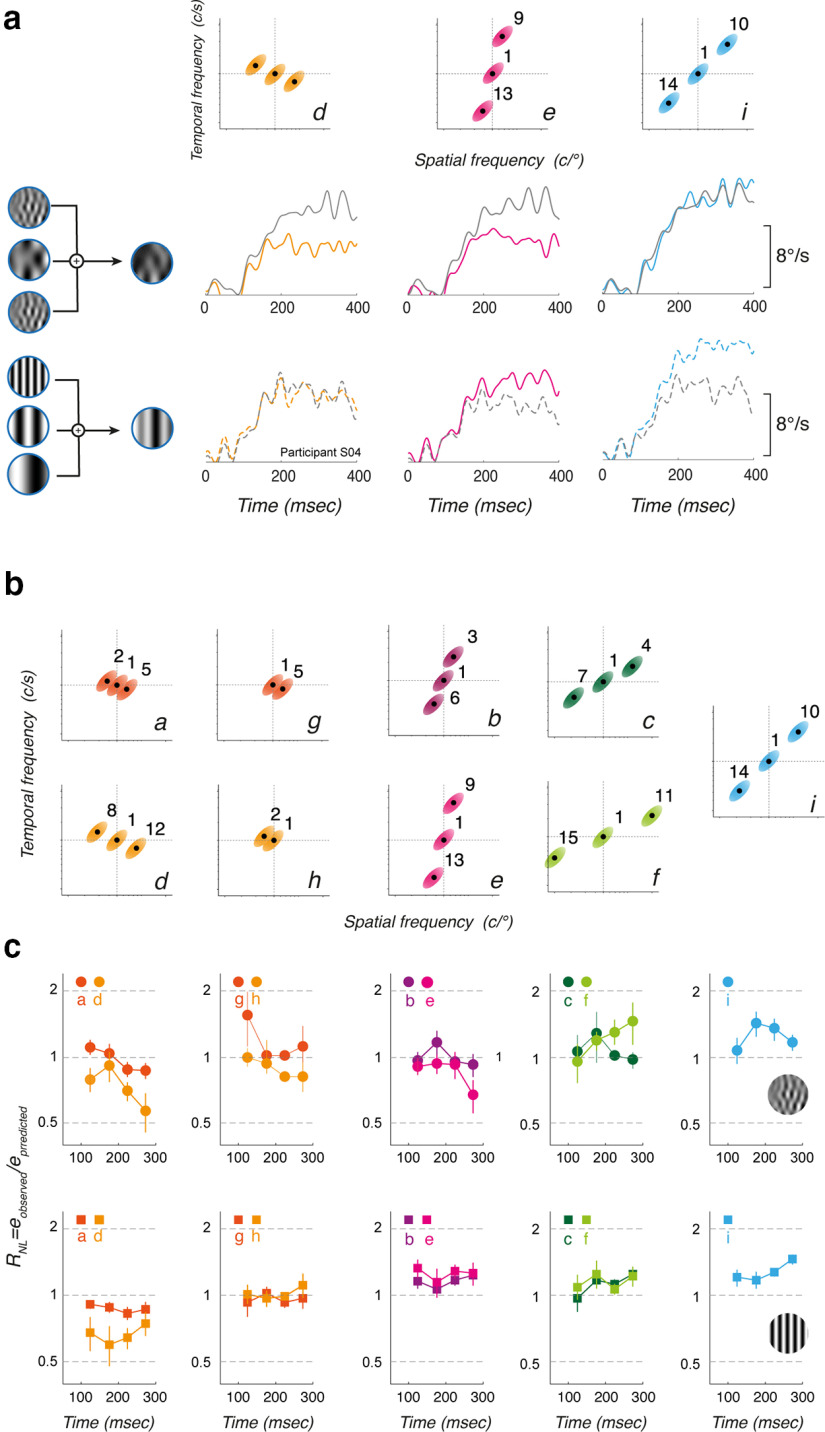
Tracking responses to pattern DGs (pDG) and MCs (pMC). ***a***, Top, Three examples of triplets, aligned along the scale axis (pattern d), the speed axis (pattern i), or in between (pattern e). Next two rows illustrate mean eye velocity profiles to these pattern MCs (middle, continuous lines) or DGs (bottom, dotted lines). Colored lines show the observed response profiles for participant S04. Gray lines show the linear prediction computed by averaging the responses to each component of the triplet. ***b***, Top and Bottom Rows, Component distributions of the 15 patterns (from a to i), in the spatiotemporal space, for both pMCs and pDGs, respectively. Different orientations and distances between components were used. ***c***, The two rows plot, for each pattern type, the ratio between observed and predicted mean eye velocities, over time. A ratio of 1 indicates that observed responses are equal to the linear prediction. Values above or below 1 indicate the observed responses are larger or smaller than predicted, respectively. Data are mean (±SD) across six participants.

Once we have fully characterized the dynamics and spatiotemporal tuning of tracking responses to either component MC or DG, we can analyze the behavioral responses to moving patterns constructed by summing two or three of these components. We constructed nine patterns using DG (pDG) and MC (pMC) components, respectively (labeled from *a* to *i*). It is critical to recall that all patterns made of symmetrical triplets have the same mean speed, but different spatiotemporal properties (Fig. 1*b*; see also Table 2). With pairs, mean spatial and temporal frequencies, as well as mean speed, are affected but only marginally (Fig. 8; Table 2). We systematically varied the orientation and distance of the triplets/pairs to map the interactions between channels located at different locations within the spatiotemporal frequency space, as we did previously for speed perception (Gekas et al., 2017). Figure 8*a* illustrates three examples of triplets made of components aligned along a scale axis (pattern *d*), along the speed axis (pattern *i*), or in between (pattern *e*). As illustrated in Figure 8*b*, we generated a total of seven triplets, with four different orientations and two distances (patterns *a–f* and *i*). We also constructed two nonsymmetrical patterns (patterns *g*, *h*) made of two component pairs, to better sample interactions along the scale axis.

**Table 2:** Stimulus parameters for the pattern MCs (pMCs) or pattern gratings (pDGs)

CMC	No of MCs (*P*)	Comp MCs	Mean *v_o_*	Rel. span	Orientation
a	3	2, 1, 5	24.000	2.00	157.52
b	3	3, 1, 6	24.000	4.83	67.50
c	3	4, 1, 7	24.000	6.27	27.86
d	3	8, 1, 12	24.000	4.00	157.49
e	3	9, 1, 13	24.000	7.31	75.36
f	3	11, 1, 15	24.000	10.07	23.48
g	2	1, 5	20.108	1.00	157.52
h	2	2, 1	29.761	1.00	157.52
i	3	10, 1, 14	24.000	7.84	45.00

These five stimulus characteristics are: the total number (*P*) and identities of the individual components based on Table 1 and Figure 1, the mean speed v_o_ which is 24°/s for most stimuli except g and h, the relative Euclidean span covered by the stimulus across the frequency space in log units and the orientation angle (°) relative to the horizontal of the given stimulus in the frequency space

**Table 3:** Parameters of the model. Bolded parameters are allowed to vary to match the experimental data

Symbol	Value	Description
$\sigma_{x}$	0.5	Channel variance over spatial frequency
$\sigma_{t}$	0.5	Channel variance over temporal frequency
ρ	0.6	Correlation coefficient
g	199 ± 58.7	Channel gain multiplier (unique for each participant)
$w_{i}$	From polynomial	Weight of channel $\varphi_{i}$
$b_{0-5}$	[−23.88, −3.63, 1.95, −0.72, 0.61, −0.45]	Polynomial parameters of channel weights
α	2.16	Interaction weight
$\sigma_{e1}$	0.2542	Excitation SD over *x*-axis
$\sigma_{e2}$	0.7718	Excitation SD over *y*-axis
$\sigma_{i1}$	0.2501	Inhibition SD over *x*-axis
$\sigma_{i2}$	0.7711	Inhibition SD over *y*-axis
θ	3π/4	Rotation of the interaction profile
$\mu_{prior}$	2.51 ± 1.35	Mean of prior distribution (unique for each participant)
$\sigma_{prior}$	0.86 ± 0.36	SD of prior distribution (unique for each participant)

One classic, basic observation about motion integration for tracking initiation is that a linear sum/average of all motion inputs can be quickly computed (Ferrera and Lisberger, 1997; Mestre and Masson, 1997; Masson and Castet, 2002). Therefore, we tested whether pattern-driven responses may be predicted from component-driven ones. One simple, linear assumption is that responses to a given pattern are identical to the average of the responses to its components. Any deviation from the linear prediction would unveil interactions between channels sampling the components. We show both observed and predicted mean eye velocity profiles for three examples in Figure 8*a*. Responses to pattern motion stimuli are indicated by colored lines, each color referring to the corresponding triplet. Gray lines illustrate the theoretical eye velocity profiles, obtained by averaging the responses to each component, already illustrated for the same participant S04 in Figure 2*a*. Upper and lower rows correspond to pMC and pDG, respectively. From this participant, representative of our 5 other participants, several features can be observed. First, for both pMC and pDG, responses reach higher eye velocity when triplets are aligned along the speed axis (pattern *f*). Responses become smaller as the triplets orientation lies along the scale axis (pattern *d*). Second, observed responses are either larger or smaller than the linear prediction, depending on the triplets orientation but also the stimulus type. This could indicate that nonlinear interactions would be tuned to both speed and scale domains but also be dependent on the local statistics of the motion inputs. Third, observed and predicted eye velocity diverge at different points in time, depending on the moving pattern characteristics. Early ocular tracking phases of tracking responses always match the linear prediction while later phases diverge away from it, suggesting that the nonlinear pattern of interactions builds up over time.

To capture these different effects, we estimated the deviation between the measured responses and their linear prediction by computing a nonlinear index (*R_NL_*) defined as

(8) $$R_{NL}=e_{obs}/e_{pred},$$

where *e_obs_* and *e_pred_* are the mean eye velocity over a given time window of the observed responses to the pattern and the predicted responses, respectively. When *R_NL_* > 1, observed responses are larger than predicted from a simple averaging of the component-driven responses. When *R_NL_* < 1, the pattern-driven responses are smaller than predicted. We computed *R_NL_* for all successive time windows and mean values (±SD across six participants) are plotted against time in Figure 8*c* for all nine patterns.

The pattern of interactions is remarkably similar across participants. Figure 8*c*, upper row, plots the time course of $R_{NL}$ for pMC. First, when components are aligned along (or close to) the scale axis, responses are smaller than predicted and such a reduction is larger for both later time windows and more distant components (compare patterns *a* and *d*). This later effect is confirmed when using pairs of components (patterns *g* and *h*), close to the mean speed axis. Second, when components are distributed along the speed axis (pattern *i*) or close to it (patterns *c* and *f*), responses are overall larger than linearly predicted. Such enhancements tend to be larger when components are more distant from each other, that is when they cover a broader range of spatial and temporal frequency channels along the iso-velocity line. Third, when components are distributed along a nearly vertical, iso-spatial frequency axis, responses are still larger than expected although with a smaller gain.

Comparison between top and bottom rows in Figure 8*c* shows that this pattern of interaction is different between component DG and MC. First, inhibitory interactions when components are aligned along the scale axis (patterns *a*, *d*) are stronger with pDG than pMC. Second, excitatory interactions are also stronger with pattern DG when triplets are aligned along the speed axis (pattern *i*). This gain modulation may be explained considering that components are either point-like (DG) or oriented (MC) along an iso-velocity line. Thus, the boost expected when aligning three point-like inputs along the speed axis is larger than with component MC which are already oriented along the same axis. In the same vein, these oriented inputs drive stronger responses when presented in isolation, such that when combined along the scale axis, the inhibitory interactions are smaller than when local, point-like triplets have the same global orientation along the scale axis. Lastly, one can see that the amplitude of excitatory and inhibitory interactions is modulated when triplets or pairs are distributed with larger distances along their orientation.

We quantified the effects of various factors on the nonlinearity using statistical model *M5*. In this case the dependent variable was the ratio *R_NL_*. This was predicted by fixed factors *stp*, *tw*, and orientation angle (*oa*). This last variable (*oa*) captures the Euclidean orientation of the triplets/pairs, relative to the speed axis. The random variable was participants, with separate stimulus determined baselines. There were main effects of stimulus type (*F*_(490.9)_ = 5.90, *p* = 0.0155) and time window (*F*_(527)_ = 6.90, *p* = 0.0088). Orientation angle was however not significant (*F*_(527)_ = 2.9815, *p* = 0.085). There was a significant interaction of stimulus type and time window (*F*_(527)_ = 4.57, *p* = 0.033), while interactions between stimulus type and orientation angle, and time window and orientation angle were both not significant. These statistics support some of the trends visualized in Figure 8*c*, showing that nonlinearities emerge over time, with a significant difference in the nonlinearity for the pattern MC and DG. The relationship between the response linearity and stimulus orientation angle, *oa*, which appears to change from sublinear to supralinear across the cases from left to right in Figure 8*c* is however not statistically significant and may depend on stimulus component distances from the central component and as such should be explored further in the subsequent modeling.

### Temporal dynamics of nonlinear interactions

**Figure 9.**
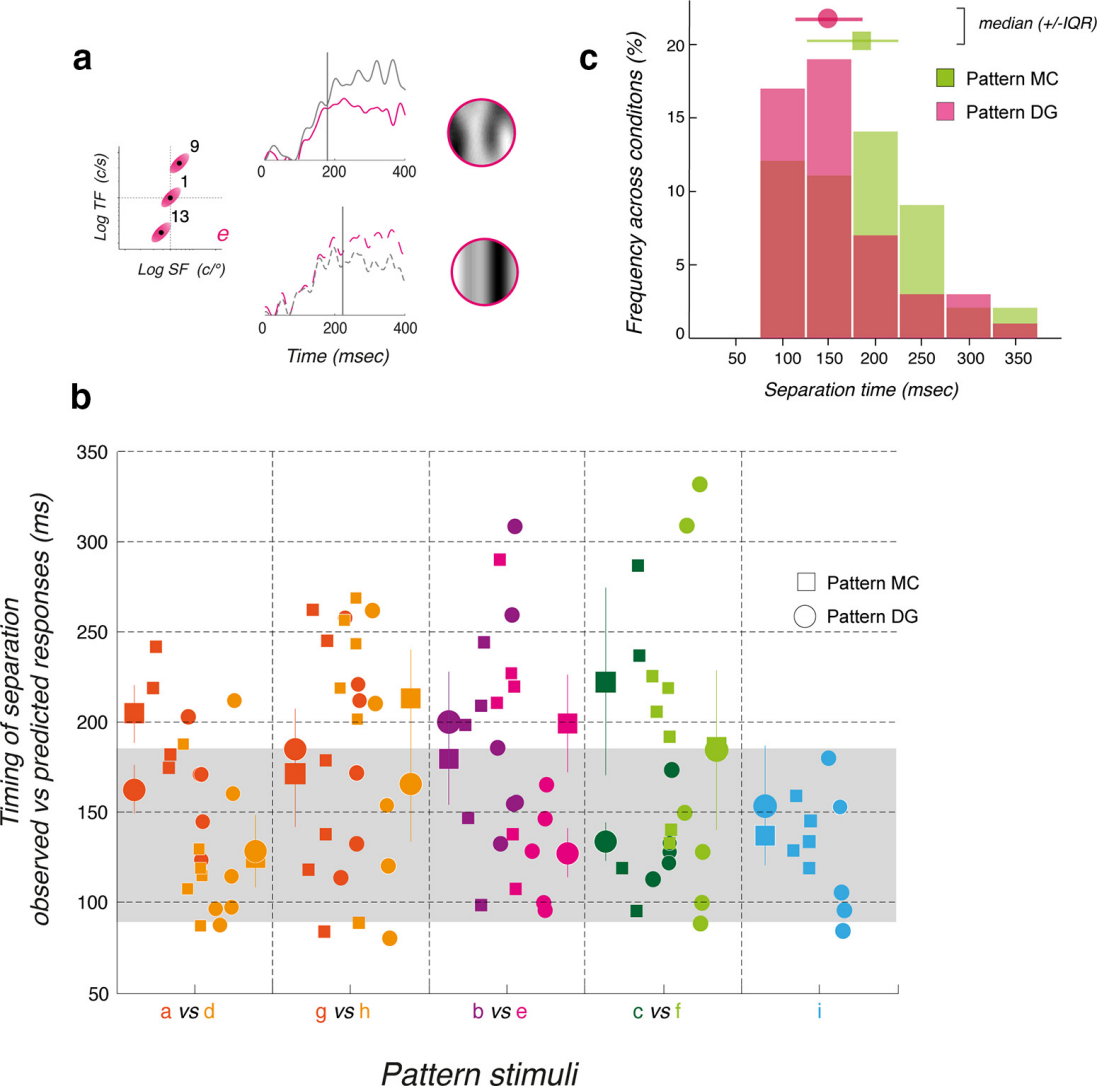
Latency of nonlinear effects. ***a***, Two examples of tracking responses for one representative participant, for pattern e composed of three components, either MC (upper plot) or DG (lower plot). Gray lines plot the theoretical eye velocity profiles obtained by averaging the tracking responses to each component, independently. Vertical dotted lines indicate the first point in time (target motion onset) at which observed and predicted tracking responses are significantly different. ***b***, The time of separation between predicted and observed eye velocity profile is plotted for each participant. For comparison, similar patterns MC (square) and DG (circle) are shown together. Larger symbols with error bars are mean (±SD) across six participants for one given condition. The gray shaded area indicates the mean open-loop period of tracking responses. ***c***, Distributions of separation times, across nine patterns and six participants, for both pattern DG (red) and MC (green) conditions. Closed red circles and green squares plot the median values (±IQR, that is the interquartile range) of separation times.

Examples of velocity profiles in Figure 8*a* and time courses of nonlinear effects in Figure 8*c* both suggest that tracking responses to patterns diverge away from their linear predictions only several dozens of milliseconds after pursuit onset and that the underlying nonlinear interactions build up over time. We first estimated when the difference between the mean eye velocity profiles of observed and predicted responses became statistically significant (Fig. 9*a*). To do so, mean and SD of observed and predicted eye velocity profiles were binned into 5-ms time windows and tested one against each other with a *t* test. Figure 9*a*, vertical dotted lines, illustrates these statistical time points of separation.

Figure 9*b* plots, for each pattern, the individual estimate of the first time point of statistical divergence between ocular tracking to patterns and the linear predictions from their respective component-driven responses. For a given pattern, square and circle symbols correspond to pattern MCs and DGs, respectively. Large symbols indicate the mean (±SD) values across participants. Overall, responses diverged between 150 and 200 ms after stimulus onset, that is between 50 and 100 ms after tracking response onset. No systematic differences between stimulus types were observed. Average (mean ± SD across participants) time of divergence was 161 ± 70 ms across all pattern DG and 185 ± 70 ms across all pattern MC (Fig. 9*c*, circle and square symbols). Figure 9*c* plots the distributions of separation times, across six participants and nine patterns. These distributions exhibit atypical skew and kurtosis characteristics (median and interquartile range: 149 and 72 for pattern DG; 185 and 98 for pattern MC). With our last statistical model, *M6*, we conducted a nonparametric, bootstrapped *t* test with 1000 runs of a trimmed mean estimation of separation onset against stimulus type (*stp*). The difference between the separation onset times of the pattern MCs and DG traces was not found to be significant (*t*_(29)_ = 1.93, *p* = 0.063). Thus, the temporal dynamics of nonlinear interactions is approximately constant across stimulus types. Since the latency of pattern-driven tracking responses were 80.6 ± 5 and 89.3 ± 9.5 for pattern DG and MC, respectively, one can conclude that most separation times occurred during the open-loop period of pursuit (that is from ∼90 to ∼180 ms after stimulus onset). Thus, nonlinear interactions are because of sensory processing and not a mere effect of tracking responses on retinal image motion. This is also supported by the fact that eye velocity reached only a fraction (∼8–10°/s) of stimulus speed (24°/s) at the end of our 400 ms of measurement after stimulus motion onset.

### Linear and nonlinear interactions shape motion computation for ocular tracking

The results presented above highlight four main properties of motion processing for ocular tracking. First, the optimal range of spatial and temporal frequencies to drive tracking response is different for DGs and MCs and change over time for the latter, not the former. Second, tracking responses to DG are tuned for both spatial and temporal frequencies. By consequence, the tuning curves are best described by a scale dimension, where scale is the product of spatial and temporal frequency. By contrast, tracking responses to MC become tuned for input speed, being less sensitive to its mean spatial and temporal frequency or, equivalently, its scale. Third, tracking responses to patterns made of several components can be either suppressed or enhanced depending on the orientation of the pattern. When the pattern is aligned along the speed axis, the observed response is larger than the linear (average of the component-driven responses) prediction. When the pattern is rotated away from the speed axis and toward the scale axis, the pattern-driven responses are smaller than linearly predicted. Fourth, responses deviated from the linear prediction only ∼100 ms after tracking initiation, unveiling the temporal dynamics of linear-nonlinear interactions. These results suggest that, after 100 ms, ocular tracking is driven by a nonlinear estimation of target speeds that takes into account the stimulus statistics. We attempted to model this observed set of properties with a dynamic probabilistic model.

### Modeling the dynamics of speed estimation

Following our original approach for speed discrimination (Simoncini et al., 2012; Gekas et al., 2017), we present here a probabilistic model to simulate the diverse set of results and infer the pattern of interactions that shape speed processing for both simple and complex retinal moving images. In a nutshell, the model is based on four stages. First, the stimulus is processed by a bank of spatiotemporal channels that can represent speed as various combinations of spatial and temporal frequencies. Each stimulus is filtered by this bank of channels, thereby simulating a neural population activity. Second, the channels interact with each other, and the interaction pattern depends on the population activity. Third, speed is read out of the population activity by applying an optimal likelihood decoder. Finally, in the fourth stage of the model, estimated speed is combined with prior temporal estimates. By taking into account the history of such estimates, it has been shown that one can simulate the temporal evolution of eye velocity during pursuit responses (Montagnini et al., 2007; Bogadhi et al., 2011).

The first stage of the model consists of spatiotemporal channels defined as bivariate normal distributions in Fourier space so that:

(9) $$\varphi_{i}\left(f_{x},f_{t}\right)∼N(\mu ,\Sigma ),$$

where

(10) $$\mu =\left(\frac{f_{x,i}}{f_{t,i}}\right)and\Sigma =\left(\sigma_{x}^{2}\rho \sigma_{x}\sigma_{t}\rho \sigma_{x}\sigma_{t}\sigma_{t}^{2}\right).$$

**Figure 10.**
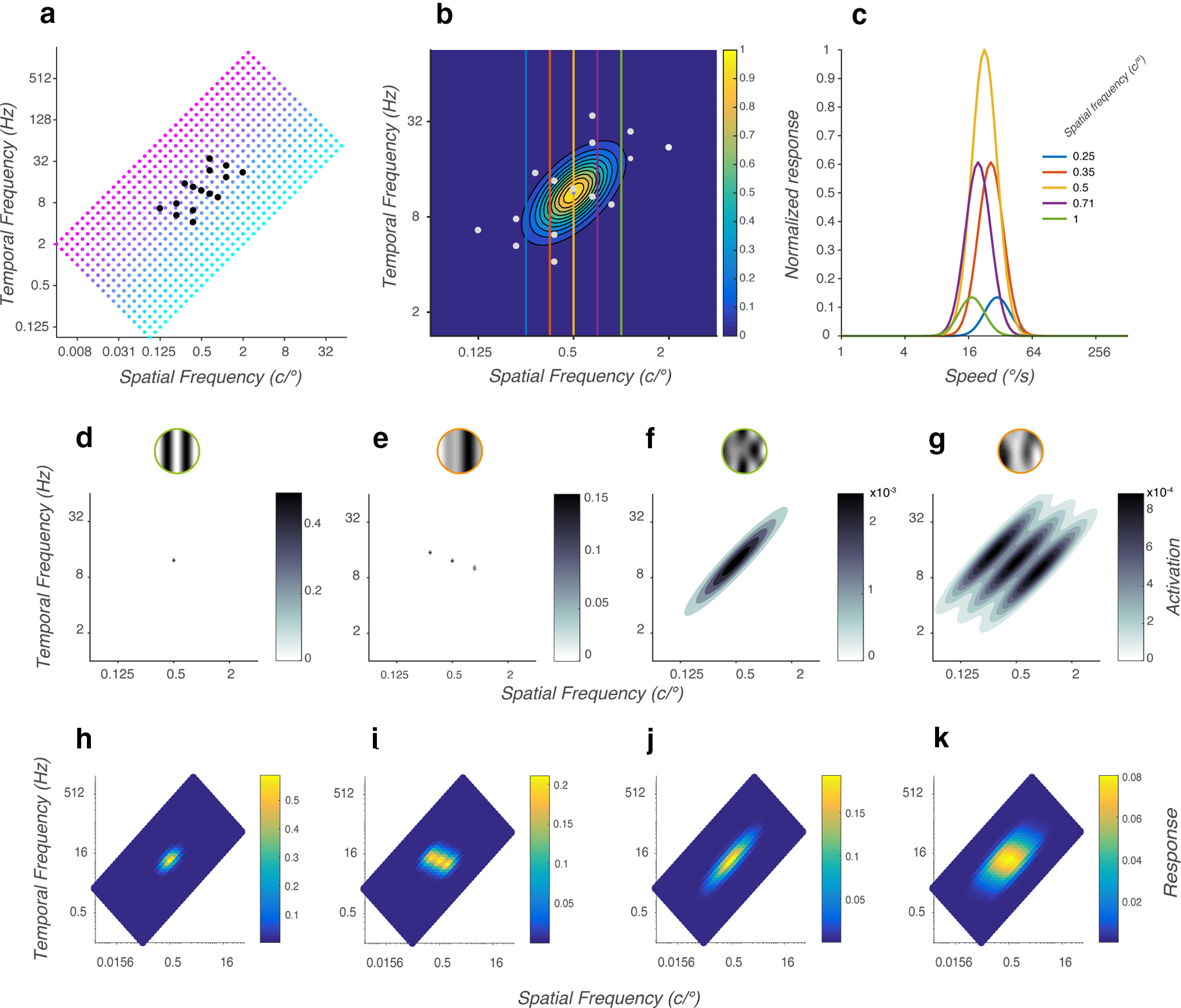
Model. ***a***, Tiling of spatiotemporal channels. Each channel is a bivariate normal function with its main axis oriented along a line of constant speed. All channels coding for the same speed are shown by the same color: blue for slow speed and pink for fast speed. The colored dots represent the center of the channels. The black dots represent the coordinates of the component stimuli. ***b***, Receptive field of a channel. The contour lines show the receptive field in log-log Fourier space of a channel centered at 0.5 c/° and 11.31 Hz. The white dots represent the coordinates of the component stimuli. ***c***, Speed tuning of a channel. Each curve shows the speed tuning of the channel in ***b*** at one spatial frequency; different colors indicate different spatial frequencies and the colors of the curves match the vertical lines in ***b*. *d–g***, Representation of different stimuli types in the log-log Fourier space. Component DG 1 (***d***), PDG d (***e***), component MC 1 (***f***), and PMC d (***g***). ***h–k***, Normalized responses of all channels of the network to the respective stimuli of ***d–g***.

Each channel is determined by its location (central spatial $f_{x,i}$ and temporal $f_{t,i}$ frequencies), spread (variance in spatial $\sigma_{x}$ and temporal $\sigma_{t}$ frequencies), and a correlation coefficient ρ=0.6 that determines the concentration along the diagonal line of constant velocity (a correlation of 1 stands for pure speed channels whereas a correlation of 0 stands for completely separable channels in spatial and temporal frequencies). The channels (N=722) are homogeneously distributed in a rectangle from 1°/s to 512°/s preferred speed (Fig. 10*a*), thereby covering the full window of visibility of human motion sensitivity and more).

For a stimulus s with contrast c each channel $\varphi_{i}$ produces response:

(11) $$m_{i}\left(s,c\right)=g2^{w_{i}}{{\int \int }^{}}_{}^{}\varphi_{i}\left(f_{x},f_{t}\right)*s(c)df_{x}df_{t},$$

where g is a gain parameter for each participant, and $w_{i}$ is the channel weight. Low channel weights correspond to channels that are beyond the window of visibility, whereas high channel weights correspond to the peak speed sensitivities. Channels are fixed across time and are assumed to account for characteristics of the network such as density of neurons for different spatiotemporal frequencies or strength of connection to the next stage. The weight of each channel is assumed to follow a quadratic bivariate polynomial surface that depends on the central spatial and temporal frequency of the channel:

(12) $$w_{i}=b_{0}+b_{1}f_{x,i}+b_{2}f_{t,i}+b_{3}f_{x,i}^{2}+b_{4}f_{x,i}f_{t,i}+b_{5}f_{t,i}^{2}.$$

In the second stage of the model, we introduce time-dependent interactions between the channels. At each time window ω, we assume dynamic patterns of interactions $I_{i}^{\omega }$ between channels so that the activity of the network is:

(13) $$n_{i}^{\omega }=m_{i}I_{i}^{\omega },$$

where the interaction depends on the activity of the network. We assume a mechanism in which channels interact with each other based on their relative distance $d_{ij}$ in spatiotemporal frequency space:

(14) $$I_{i}^{\omega }=\overset{N}{\underset{j=1}{\sum }}m_{j}^{\omega }d_{ij}.$$

The activity of each channel is inceased or decreased by the weighted sum of the activity of its surrounding channels. The interaction pattern is composed of an excitatory and an inhibitory component. Each component is described as a bivariate Gaussian function over speed and scale:

(15) $$d_{ij}=\alpha \left(\frac{N_{exc}(\mu_{exc},\Sigma_{exc})}{max(N_{exc}\left(\mu_{exc},\Sigma_{exc}\right))}-\frac{N_{inh}(\mu_{inh},\Sigma_{inh})}{max(N_{inh}\left(\mu_{inh},\Sigma_{inh}\right))}\right),$$

where

(16) $$\mu_{exc}=\left(\frac{0}{0}\right),\Sigma_{exc}=\left[cos(\theta )sin(\theta )-sin(\theta )cos(\theta )\right]\left[\sigma_{e1}^{2}00\sigma_{e2}^{2}\right]\left[cos(-\theta )sin(-\theta )sin(-\theta )cos(-\theta )\right]$$

(17) $$\mu_{inh}=\left(\frac{0}{0}\right),\Sigma_{inh}=\left[cos(\theta )sin(\theta )-sin(\theta )cos(\theta )\right]\left[\sigma_{i1}^{2}00\sigma_{i2}^{2}\right]\left[cos(-\theta )sin(-\theta )sin(-\theta )cos(-\theta )\right],$$

α is a magnitude parameter, $\sigma_{e1}^{2},\sigma_{e2}^{2},\sigma_{i1}^{2},\sigma_{i2}^{2}$ are the variances in octaves, and θ=3π/4. The same interaction pattern is applied to each channel regardless of its mean spatial and temporal frequency and across time.

In the third stage of the model, the logarithm speed likelihood of each channel is computed:

(18) $$log\;p\left(s\right)=n_{i}^{\omega }log(\psi_{i}),$$

where $\psi_{i}$ is the speed tuning of channel i centered on speed $v_{i}=\frac{f_{t,i}}{f_{x,i}}$. The speed tuning function of a channel that is a bivariate normal distribution is the cross-section along the $f_{t}=-f_{x}$ axis:

(19) $$\psi_{i}\left(v\right)=e^{-\frac{{\left(v-v_{i}\right)}^{2}}{2\sigma^{2}(1-\rho )}}.$$

The log-likelihoods are then summed across all channels to give the overall speed log-likelihood of the stimulus:

(20) $$log{\left(L\left(s\right)\right)}^{\omega }=\overset{N}{\underset{i=1}{\sum }}n_{i}^{\omega }log(\psi_{i}).$$

In the fourth stage of the model, we take into account the history of the stimulus processing in the network. A posterior probability of speed at time window ω is taken as the combination of the likelihood and prior at this time window ω. The prior at time window ω is the posterior of stimulus at the previous time window ω−1, so that the posterior distribution at ω is:

(21) $$posterior{\left(s\right)}^{\omega }={\left(L\left(s\right)\right)}^{\omega }posterior{\left(s\right)}^{\omega -1}.$$

The posterior at the first time window ω=0 (i.e., the initial prior distribution) is assumed to be a lognormal distribution with mean $\mu_{prior}$ and SD $\sigma_{prior}$. We find the model parameters M (summarized in Table 3) that maximize the log-likelihood of matching the experimental OFRs with the model for all stimuli at each time window:

(22) $$M=argmax_{M}\left[\overset{S;\Omega }{\underset{s=1;\omega =1}{\sum }}log(p\left(s\right))\right].$$

We consider the window between 100 and 150 ms as the first time window (ω=0), and we fit the parameters of the model to the following three time windows 150–200, 200–250, and 250–300 ms.

There are four types of stimuli: component DGs, pattern DGs (pDGs), component MCs, and pattern MCs (pMC; Fig. 10*d–g*). DGs are defined as points in the log-log spatiotemporal frequency space and the response of each channel to the grating is computed from the value of the tuning function at the coordinates of the stimulus multiplied by the contrast. pDGs are defined as the averaged activation to their corresponding component DGs. MCs are defined as ellipses in frequency space so they activate multiple points with different intensity (see MC equation). pMCs are defined as the averaged response to their corresponding component MCs. The response of each channel is calculated as the weighted sum of the responses to each point in the discretized frequency space (Fig. 10*h–k*). For DGs, this is calculated directly from the bivariate normal distribution of the channel. For MCs, the channel response is calculated as the weighted average of a large number of individual gratings.

### Model fitting

**Figure 11.**
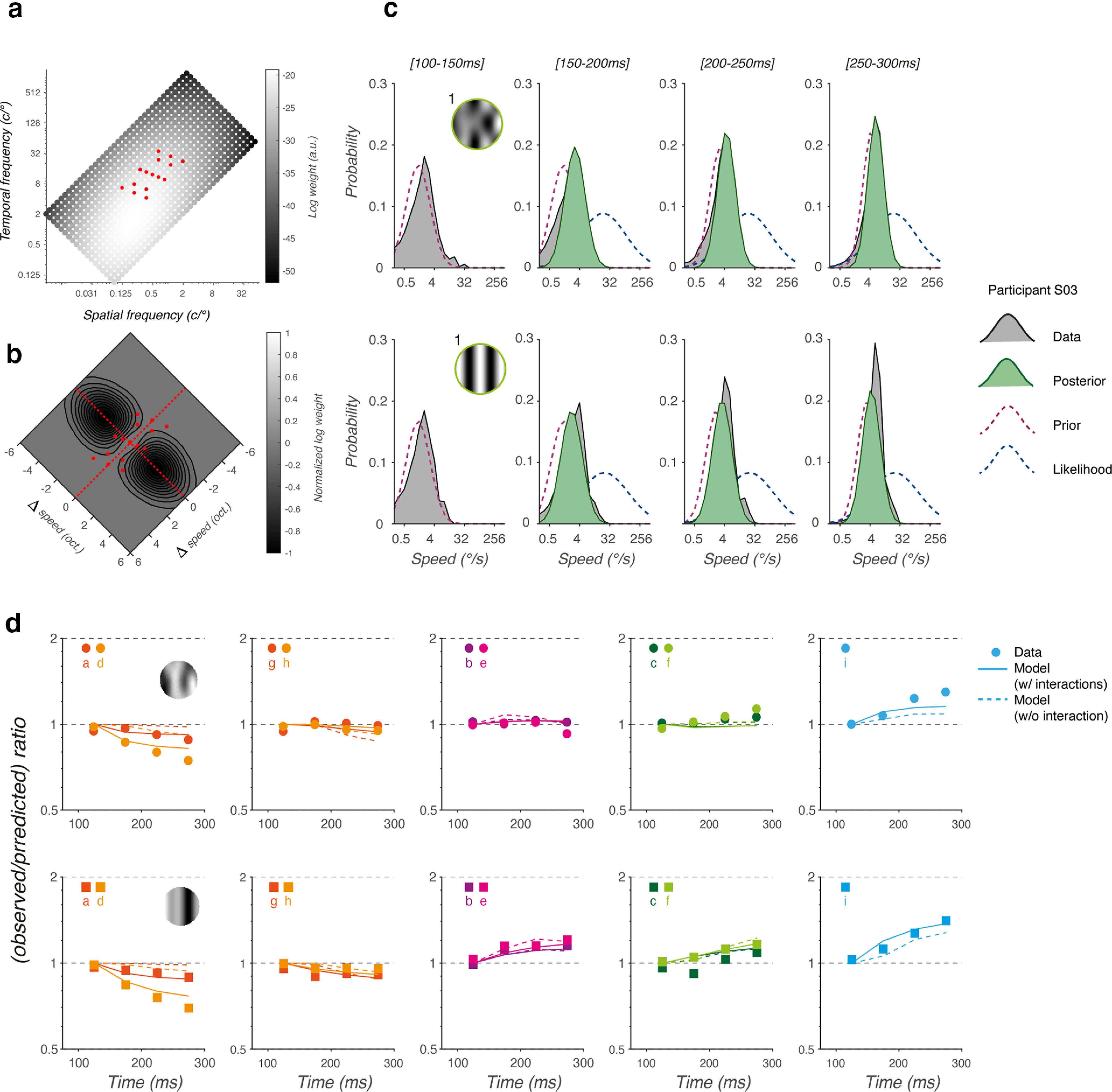
Model performance. ***a***, Distribution of channel weights across the network. ***b***, Best-fit interaction pattern. ***c***, Comparison of responses probabilities across time windows between experimental and model data, for one participant (S03). ***d***, Ratios between observed and predicted mean eye velocities (plotted on a Log scale) to pattern stimuli over time plotted for experimental data, the model with interaction and the model without interaction. Colors and labels are identical to Figure 6.

**Figure 12.**
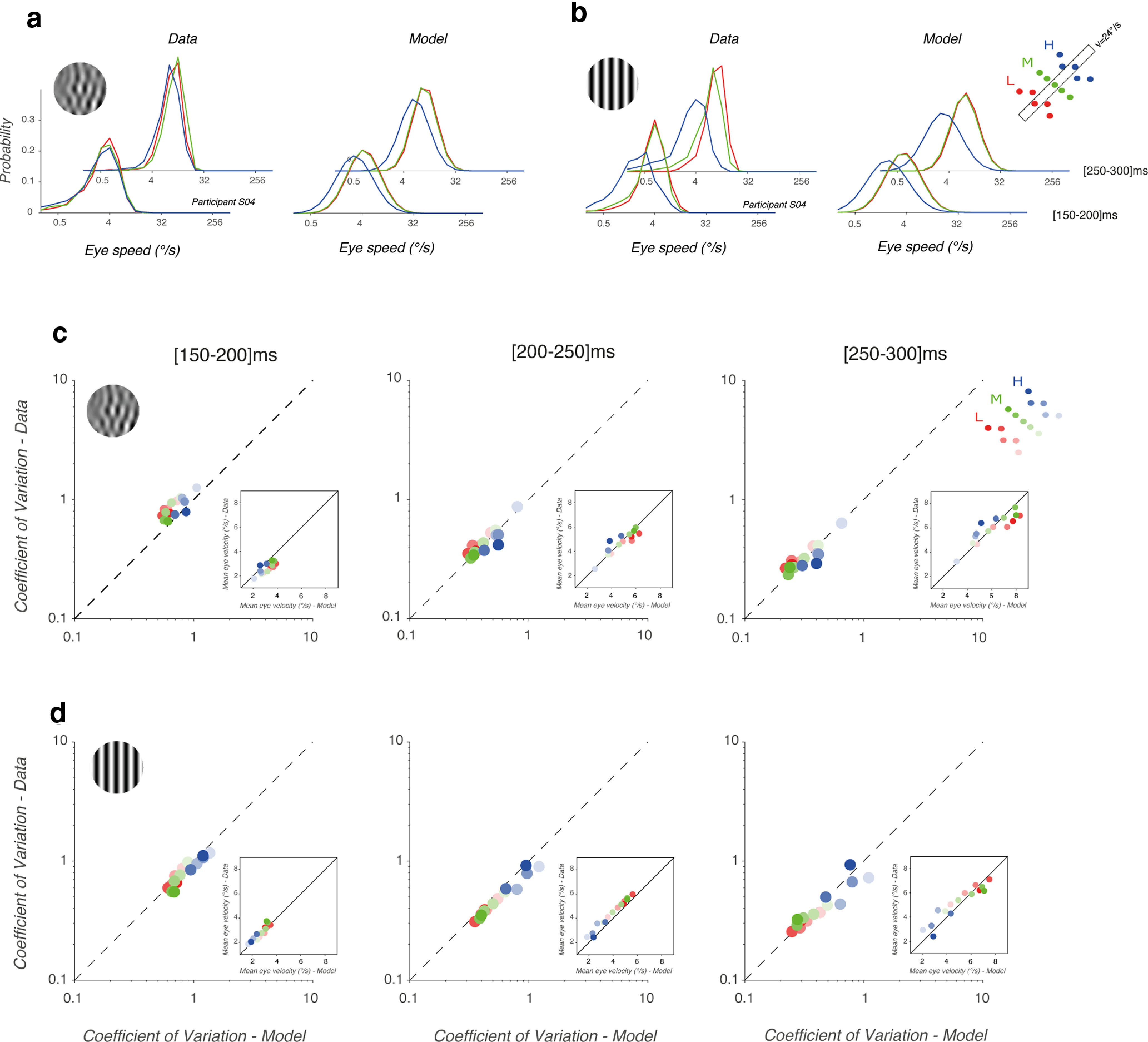
Comparison between data and model for component stimuli. ***a***, Distribution of observed (data) and predicted (model) eye velocity of ocular responses to a component MC moving at 24°/s, for participant S04. Color indicates scale range. First and second row illustrate early ([150–200 ms]) and late ([250–300 ms]) time windows. ***b***, Same plots, but now for component DG of the same mean spatial and temporal frequency and mean speed (24°/s). ***c***, ***d***, Relationship between predicted and observed CV (coefficient of variation) across the three scales (color) and five speeds (saturation), for three windows. Insets plot the same relationships between model and data but now for mean eye velocities. Oblique lines indicate a slope of 1 for the linear regression.

The model attempts to capture not only the differences in OFRs between different types of stimuli (DGs and MCs) but also stimuli that differ in their spatiotemporal characteristics. To accomplish that, it assumes that channels centered at different spatiotemporal coordinates have distinct weights. So, stimuli that activate different channels will produce larger or smaller responses depending on the weight of these channels. The weights predicted by the model are plotted in Figure 11*a*. Weights are maximized at channels centered on 0.21 c/^o^ and 1.68 Hz and decrease further away from these coordinates. Furthermore, the model is able to track the dynamics of the OFR at different time windows. Figure 11*c* shows two examples of the evolution of the response likelihood for one example participant (S03) for the central components DG *c1* and MC *c1*. At each time window, the speed likelihood (blue dashed curves) produced by the network is combined with the prior probability (red dashed lines) to derive the posterior probability (green curves) of response. The close similarity of the posterior with the participant’s responses (gray curves) shows that the model can capture the mean eye velocity and the variance in responses across trials. A comparison of mean eye velocities to component DG and MC between the model and the data are shown in Figure 12. Figure 12*a*,*b* plots the distributions of observed and simulated distributions of eye velocity at two different time windows. For a component MC moving at 24°/s (see also Fig. 3*a*), varying input scale has very little impact on both the mean and width of these distributions. On the contrary, both data and model distributions for a single DG moving at the same speed show that at higher scale (blue curves), responses become slower (a shift in the mean) and more variable (an increase in width). Similar differences were observed at lower (11°/s and 16°/s) and higher speeds (36°/s and 53°/s). This result illustrates that with MC, the model tuning is scale invariant for MC, but not for DG. The model is also able to capture the different evolution of responses to either single MC or DG over time. There is a high correlation between predicted and observed mean eye velocities at different time scales (Fig. 12*c*,*d*, insets), both at group (*r*^2^ = 0.89) and individual levels (0.90 > *r*^2^ > 0.20). Moreover, predicted responses to single DGs at the highest scale were also much smaller than for single MCs. The model can also capture the relative variabilities of responses to either DG or MC, and their dynamics. Over time, the normalized variability (i.e., the CV, *r*^2^ = 0.80) decreases for both model and human responses to MC, regardless of stimulus scale (Fig. 12*c*,*d*). By comparison, both predicted and observed CV remains constant over time with DGs, in particular because of the larger variability and smaller responses seen with single DGs at high scale.

Importantly, the model assumes a pattern of interaction between channels depending on their relation in the spatiotemporal space or in speed-scale coordinates (Fig. 11*b* and model compared with example component data in Fig. 12). The simulated pattern indicates strong inhibition between channels along the same scale that is maximized approximately at a distance of three octaves and decreases for orientations away from the scale axis. Channels that are along the same speed lines show minimal or weakly positive interactions. This interaction is required to explain the systematic deviations from the linear predictions exhibited by the pattern stimuli. Figure 11*d* shows a comparison of ratios between observed and predicted mean eye velocities for each pattern stimulus for the MCs and DGs and the fits of the model with (solid curves) and without (dashed curves) interaction. While the model without interaction can predict some of the smaller deviations found in patterns with components between the scale and speed axis, it cannot explain the strong sublinear and supralinear responses to pattern stimuli with components along the scale and the speed axis, respectively.

**Figure 13.**
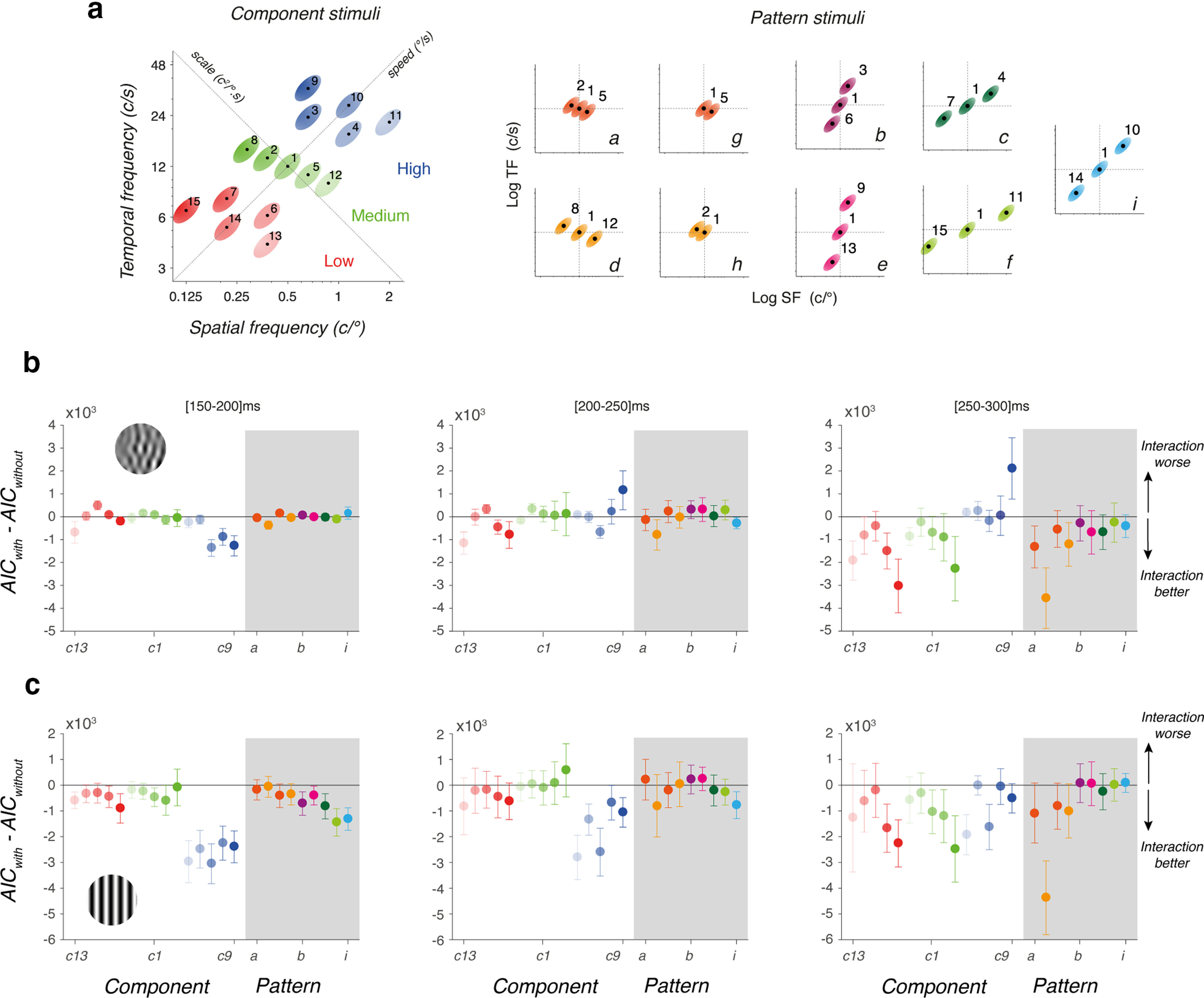
Comparison between linear and nonlinear models of motion integration for speed estimation. AIC values were computed for each model and component or pattern conditions and each participant. The mean (±SD across participants) difference between AIC_with_ and AIC_without_ values is plotted for all 15 components (c1 to c15) and nine patterns (from a to i). Positive and negative values indicate that the nonlinear, interaction model is worse or better than a model without interactions, respectively. ***a***, Color codes for either component (left) or pattern (right) stimuli. ***b***, Component and pattern MC. ***c***, Component and pattern DG.

To evaluate the quality of our model, we computed the Akaike Information Criterion (AIC) for the model fits to individual data, with (AIC_with_) or without (AIC_without_) the interaction pattern. Figure 13 plots the mean differences between the two models. Since the preferred model is the one with the minimum AIC value, positive and negative differences indicate that the model with interactions performs worse or better than without. By plotting this index for both component and pattern DG/MC at different time windows, it is possible to track when the interactions between spatiotemporal channels become necessary to fit the data. Indeed, with both component and pattern MC, both models fit the data at the first two time windows equally well but with the latest time window, the interaction model outperforms, for almost all conditions except the component with the highest scale range. The signal level on these high scale responses is generally lower than the rest of the range. With pattern MC and DG, early eye velocity is best fitted with interactions, in particular the inhibitory interactions along the scale axis (patterns *a*, *d*, *g*, *h*).

## Discussion

We investigated the spatiotemporal tuning of reflexive ocular tracking responses in humans, using both DGs and moving textures. We used these responses to predict tracking eye movements to patterns made of either two or three of these component motions. Altogether, our results document both spatiotemporal frequency tuning for simple and complex inputs and the dynamic interactions underlying speed representation.

### Constant spatiotemporal tuning of grating ocular responses

Using large, moving stimuli, Miles et al. (1986) first reported in monkeys that initial eye velocity is tuned for low spatial frequencies, with a peak at ∼0.3 c/°, and mid-range temporal frequencies, with a peak at ∼10 Hz. Similar tuning was subsequently reported in humans (Gellman et al., 1990; Sheliga et al., 2005; Quaia et al., 2012). In both human and nonhuman primates ocular following, the spatiotemporal tuning envelope orientation is biased toward the temporal frequency axis and is mostly separable into spatial and temporal frequency tuning (Hayashi et al., 2010; Miura et al., 2014; Sheliga et al., 2016). Interestingly, in macaque monkeys, ocular following spatiotemporal tuning resembles the weighted sum of the envelopes describing the neural population tuning of MST and MT (Miura et al., 2014).

With DGs, our results are consistent with previous findings. First, both early and late eye velocities are best tuned for spatial and temporal frequency, over the range of tested frequencies. We found a nearly symmetric envelope peaking at low spatial (∼0.3 c/°) and mid temporal (∼10 Hz) frequencies. For the first time we reconstructed the temporal dynamics of spatiotemporal tuning and showed that it remains constant over time, despite the large change in retinal image motion occurring over the first 300 ms of tracking. This tuning is consistent with previous studies in humans (Hayashi et al., 2010; Sheliga et al., 2016) and monkeys (Miura et al., 2006) and might reflect the contribution of temporal frequency tuned neurons found in monkey area MT when tested with gratings (Perrone and Thiele, 2001; Priebe et al., 2003; Miura et al., 2006; Zaharia et al., 2019). We propose that the scale dimension, defined as the product of spatial and temporal frequencies, best summarizes this constant spatiotemporal tuning of OFRs to gratings.

### Orientation of local motion energy improves speed tuning

Spatiotemporal tuning for perception (Kelly, 1979) and ocular tracking (Hayashi et al., 2010) was primarily previously probed with drifting sinusoidal gratings, that are point-like (nonoriented) motion stimuli in Fourier space. Here, we systematically compared gratings with broadband oriented MCs (Sanz-Leon et al., 2012; Vacher et al.,2018). While DG have zero variance, MCs have independent variance along spatial and temporal frequency. We constructed them by constraining MC spatiotemporal distributions to be oriented along a speed axis unlike DG, while both stimuli retain matched mean speed, spatial and temporal frequencies and contrast energy. We contrasted their response patterns. First, oriented MC stimuli systematically elicited stronger tracking velocity, in particular from ∼100 ms after response onset, with higher reliability across trials. These results are consistent with our earlier finding that broadband energy distributions along a given iso-velocity line result in stronger and more reliable ocular following (Simoncini et al., 2012). Speed tuning of MCs responses was constant over time, with an optimal speed range of 30–50°/s, consistent with human OFRs to either DGs (Gellman et al., 1990), random dot (Quaia et al., 2012), or 1D pink noise patterns (Sheliga et al., 2020). Second, for DG the reduction in eye velocity was stronger for the highest scale components, corresponding to high spatial and temporal frequencies. In contrast, MCs enlarge the optimal range of spatiotemporal frequencies for decoding input speeds so that speed tuning becomes independent of spatial and temporal features (scale invariance) over the tested range and the 2D spatiotemporal envelope becomes elongated along a speed axis in Fourier space (reflecting inseparability).

These results are consistent with several properties of cortical speed tuned neurons. In areas V1 and MT, few direction-selective cells are truly speed-tuned (Perrone and Thiele, 2001; Priebe et al., 2003, 2006). When tested with broadband motion stimuli such as random dot patterns, speed tuning is sharper and responses are stronger than with DGs (Priebe et al., 2003; Solomon et al., 2010). Moreover, pattern-selective MT cells in macaques and marmoset monkeys prefer higher speeds (Priebe et al., 2003; Krekelberg et al., 2006; Solomon et al., 2010; Wang and Movshon, 2016) and tend to be better described as spatial frequency inseparable, or equivalently “velocity-separable” (Zaharia et al., 2019). Such properties might emerge from recurrent interactions, in particular excitation along the iso-velocity line and inhibition away from it (Inagaki et al., 2016). We propose that contrasting oriented and point-like component stimuli is an efficient approach to titrate their relative contribution. We now discuss how pattern stimuli can further unveil the nonlinearities at neuronal levels (Meso and Zanker, 2009; Meso and Simoncini, 2014; Inagaki et al., 2016; Zaharia et al., 2019).

### Decoding pattern speed is shaped by nonlinear interactions between frequency channels

Computational rules and neural mechanisms of motion direction integration have been largely investigated by comparing the perceptual, behavioral and neuronal responses to either component or pattern motion stimuli (for review, see Born and Bradley, 2005; Nishida et al., 2018). Only recently have some studies directly linked motion direction and speed computation (Zaharia et al., 2019). Ocular responses to patterns made of different direction components have unveiled several nonlinear properties (Priebe et al., 2001; Masson and Castet, 2002; Hayashi et al., 2010; Sheliga et al., 2020). Here, we show that early reflexive tracking depends on the orientation of the motion components relative to the speed axis. Responses to patterns made of components aligned along an iso-velocity line are always larger than predicted by averaging the corresponding component-driven responses, what we call the linear prediction. The gain is higher when components are DGs, and not oriented local components as expected since responses to single gratings were in fact smaller than to single MC. Rotating the component axis relative to this iso-velocity line reduced eye velocity and when the three components were distributed along the scale axis, responses were less than linearly predicted. Recall that moving patterns had the same mean retinal speed and so comparing response amplitudes relative to the linear (average) prediction alleviates any differences in amplitude explained by the spatiotemporal tuning of ocular tracking. Thus, positive or negative changes in tracking responses can be attributed only to interactions, excitatory or inhibitory, respectively, between channels sensing the different components.

Our model reveals this interaction pattern from the likelihoods of response amplitudes for each component and pattern input. Vector average computation cannot account for ocular responses to both pattern DG and MC. Rather, we identified two orthogonal interactions within the spatiotemporal frequency space, aligned with the scale and speed axes. Inhibition is strong between channels along the same scale and is maximized approximately at a distance of three octaves. It decreases for orientations away from the scale axis. Channels that are along the same iso-velocity line show minimal or weakly positive interactions. As expected, these positive interactions were stronger with pDG than pMC, since single MC are already oriented along the same axis. These patterns of interactions can account for the response amplitude and variability across trials.

Such a pattern of crossed speed-scale interactions is consistent with our previous results with both ocular following and speed perception (Simoncini et al., 2012; Gekas et al., 2017). It is also consistent with recent neuronal studies investigating the structure of velocity selectivity in macaque MT neurons. Inagaki et al. (2016) found that their spectral receptive field is shaped by excitatory inputs along the iso-velocity line and broad suppressive inputs. We propose a rationale for the organization of these inhibitory interactions: they are stronger along the scale axis. Such a suppressive structure would improve the reliability of velocity representation while supporting motion segmentation. We will discuss below the functional importance of such crossed excitation/inhibition patterns.

### From linear to nonlinear motion integration: temporal dynamics

A popular belief is that speed integration collapses into a single local averaging of all inputs within the central visual field (Mestre and Masson, 1997; Lisberger, 2010). There are however known nonlinearities that shape the initiation of tracking eye movements, such as contrast gain control or center-surround interactions (Barthélemy et al. 2006, 2008; Sheliga et al., 2016, 2020). Ocular following tracks the temporal dynamics of the cascade of motion processing steps from local luminance measurements to global speed/direction integration (for review, see Masson and Perrinet, 2012). Here, we unveiled three aspects of speed computation during a transition from linear to nonlinear mechanisms.

First, the earliest part of ocular tracking is tuned for speed, spatial and temporal frequencies and broadband motion inputs drive larger initial eye acceleration (Hayashi et al., 2010; Simoncini et al., 2012). Further comparison between point-like (DG) and oriented (MC) inputs reveals that a true speed representation is achieved only with oriented inputs. The speed representation is largely invariant to spatial and temporal properties over the three to four octaves studied. Conversely, responses to DG are more tuned for the spatiotemporal scale, rather than speed.

Second, the reliability of speed representation gradually builds over time for oriented MC stimuli. This result is consistent with the observation that tracking responses to moving oriented patterns with broader spatial frequency distributions are more precise and reliable than for point-like moving inputs (Simoncini et al., 2012; Mukherjee et al., 2015). Thus, distributing motion energy along an iso-velocity line in Fourier space sharpens speed representation and improves the reliability of decoding. Similar temporal dynamics was reported for smooth pursuit in monkeys, where both discrimination threshold and accuracy improves over ∼100 ms (Osborne et al., 2007). Lisberger and colleagues proposed that noise in sensory processing of visual motion direction and speed provides the major source of variation in the initiation of pursuit. For direction, such a time course of information driving pursuit is probably determined by noise in MT neuronal responses (Osborne et al., 2004; Osborne, 2011; Lisberger, 2015). Further studies should investigate the time course of precision and reliability of neural speed representation along the visual motion pathway contrasting oriented and point-like, alongside component and pattern motion inputs.

Overall, our results suggest that the earliest integration is best explained by a linear averaging of the different speed components. Nearly 80 ms after response onset, within the open-loop period, responses diverged from the linear prediction, bringing nonlinear mechanisms into play. Our dynamical model accounts for this, since after 100 ms of pursuit, eye velocity likelihoods are better simulated by the interaction model. Although very little is known about the time course of speed selectivity in the primate visual system, our results call for a closer examination of population dynamics of speed representation when probed with naturalistic, complex stimuli as introduced here. It should be noted that such temporal dynamics is consistent with that documented for 2D motion direction integration, at both behavioral (Masson et al., 2000; Masson and Castet, 2002) or neuronal levels (Pack and Born, 2001; Smith et al., 2005). With ocular following, Masson and colleagues identified a two-stage, cascaded motion computation. An early phase is driven by a linear integration of motion inputs, similar to vector averaging but a second phase starts ∼20–30 ms after pursuit onset and grows over time, rotating eye movement direction toward the true 2D pattern motion direction (Masson and Castet, 2002; Barthélemy et al., 2008). The same time course was later found for smooth pursuit in both humans and monkeys (Pack and Born, 2001; Masson and Stone, 2002) and is consistent with previous studies showing a similar delayed contribution by ∼50–100 ms of global or second-order motion speed to pursuit initiation in humans (Butzer et al., 1997) and monkeys (Priebe et al., 2001). For motion direction, several studies have shown that direction selectivity in monkey area MT follows the same time course, with component selective cells being activated before pattern-selective neurons and pattern direction selectivity gradually emerging over time (Pack and Born, 2001; Smith et al., 2005). At the computational level, such temporal dynamics has been explained by delayed higher-order motion integration mechanisms (Wilson et al., 1992; Löffler and Orbach, 1999), a slower inhibition (Medathati et al., 2017), and a gradual diffusion mechanism (Tlapale et al., 2010; Perrinet and Masson, 2012). Similar mechanisms may be involved in speed representation.

### Conclusion: a new view of visual speed computation

The present study strengthens the idea that speed representation is shaped by nonlinear interactions which are best captured within the scale-speed space (Gekas et al., 2017). Speed and scale axes relate spatial and temporal frequencies of an image by their ratio (speed) and product (scale), respectively. Speed tuned neurons are often understood as spatiotemporal inseparable filters, oriented along the speed axis. In the speed domain, we propose that, similar to the direction domain (Medathati et al., 2017), local excitation corresponds to excitatory interactions implementing integration along one iso-velocity line in Fourier space. With oriented motion stimuli, such as MC, speed tuning of these neurons is sharper and more reliable and, by consequence, the population spatiotemporal tuning is also oriented in Fourier space. Thus, behavioral performance is improved (Simoncini et al., 2012; Gekas et al., 2017). Inhibition is often attributed to channels located away from the same iso-velocity line, as for instance in MT receptive fields (Inagaki et al., 2016). We propose that the inhibition pattern is better captured along the scale axis. Our reasoning is that an iso-scale line corresponds to visual inputs distributed at different depths and spanning both spatial and temporal frequency gradients within a parallax flow field. Therefore, speed signals along this axis are different and should typically be segmented as belonging to separate objects. Overall, scale-speed interaction patterns would optimally parse the visual motion flow and support object segmentation. Optomotor responses could then exploit such interactions for tracking objects while discarding background motion information.

## Synthesis

Reviewing Editor: Christoph Michel, Universite de Geneve Decisions are customarily a result of the Reviewing Editor and the peer reviewers coming together and discussing their recommendations until a consensus is reached. When revisions are invited, a fact-based synthesis statement explaining their decision and outlining what is needed to prepare a revision will be listed below. The following reviewer(s) agreed to reveal their identity: Samuel Solomon. Note: If this manuscript was transferred from JNeurosci and a decision was made to accept the manuscript without peer review, a brief statement to this effect will instead be what is listed below.

The reviewers are satisfied with the revision. However, a last effort should be done to better direct the reader to what the authors think is the major importance of the work. One reviewer states that in the reply to reviewers the authors do this clearly and succinctly. He had hoped that something of similar clarity would also be present in the manuscript - he recommends something similar in the introduction so that the non-expert reader can understand this too.

Also, the significance statement is still written for a very high level - the sentence “The nonlinearity is characterised as excitation and inhibition acting separately across orthogonal speed and scale dimensions within a planar visual motion representation.” in particular. Please simplify

## Author Response

Decision on eNeuro eN-TNWR-0511-21X -Reviews and Responses to reviews (Decision dated 01 March 2022)

Editor message:

I am pleased to inform you that your manuscript, “Speed estimation for visual tracking emerges dynamically from nonlinear frequency interactions,” has been judged potentially suitable for publication in eNeuro, provided appropriate revisions are made. The decision was a result of the reviewers and me coming together and discussing our recommendations until a consensus was reached. I have put together a fact-based synthesis statement explaining the decision and appended it to this email.

Response: Thank you for the decision and for the synthesis statement that will guide our preparation of the revised manuscript. We agree with all the recommendations made by yourself and the reviewers, and lay out in the responses below how we have made changes to the revised manuscript to respond to the changes requested.

Synthesis of Reviews:

Significance Statement Comments for Author (Required):

The significance statement needs to be simplified and made accessible to the community.

Response: We agree that more work could go into this and respond further below.

Comments on the Visual Abstract for Author (Required):

N/A

Synthesis Statement for Author (Required):

The reviewers are satisfied with the revision. However, a last effort should be done to better direct the reader to what the authors think is the major importance of the work. One reviewer states that in the reply to reviewers the authors do this clearly and succinctly. He had hoped that something of similar clarity would also be present in the manuscript - he recommends something similar in the introduction so that the non-expert reader can understand this too.

Response: Thank you for this further emphasis on the importance of communicating our key message to the audience within the manuscript. We now take some of the clearer statements made in our previous responses to reviewers document (which sought to lay out the significance of our findings systematically) and adapt those for use in the revised introduction in p4 L113-117 and p4 L119-130. We believe this now does a better job of communicating this message.

Also, the significance statement is still written for a very high level - the sentence “The nonlinearity is characterised as excitation and inhibition acting separately across orthogonal speed and scale dimensions within a planar visual motion representation.” in particular. Please simplify

Response: Thank you for this comment and specifically highlighting an example of an offending sentence. We have completely re-worked the significance statement, trying to make it more general and less specialised, and even asking non-expert colleagues to advise us with a sense check on our revised version. We believe we have managed to produce a more accessible and usable statement which you can see on p2 of the revised manuscript.
